# Quantitative proteomics identifies redox switches for global translation modulation by mitochondrially produced reactive oxygen species

**DOI:** 10.1038/s41467-017-02694-8

**Published:** 2018-01-22

**Authors:** Ulrike Topf, Ida Suppanz, Lukasz Samluk, Lidia Wrobel, Alexander Böser, Paulina Sakowska, Bettina Knapp, Martyna K. Pietrzyk, Agnieszka Chacinska, Bettina Warscheid

**Affiliations:** 1 International Institute of Molecular and Cell Biology, 4 Ks. Trojdena Street, 02-109 Warsaw, Poland; 2 Centre of New Technologies, University of Warsaw, S. Banacha 2c, 02-097 Warsaw, Poland; 3 Faculty of Biology, Institute of Biology II, Biochemistry–Functional Proteomics, University of Freiburg, Schänzlestrasse 1, 79104 Freiburg, Germany; 4 BIOSS Centre for Biological Signalling Studies, University of Freiburg, Schänzlestrasse 18, 79104 Freiburg, Germany; 5 ZBSA Centre for Biological Systems Analysis, University of Freiburg, Habsburgerstrasse 49, 79104 Freiburg, Germany

## Abstract

The generation of reactive oxygen species (ROS) is inevitably linked to life. However, the precise role of ROS in signalling and specific targets is largely unknown. We perform a global proteomic analysis to delineate the yeast redoxome to a depth of more than 4,300 unique cysteine residues in over 2,200 proteins. Mapping of redox-active thiols in proteins exposed to exogenous or endogenous mitochondria-derived oxidative stress reveals ROS-sensitive sites in several components of the translation apparatus. Mitochondria are the major source of cellular ROS. We demonstrate that increased levels of intracellular ROS caused by dysfunctional mitochondria serve as a signal to attenuate global protein synthesis. Hence, we propose a universal mechanism that controls protein synthesis by inducing reversible changes in the translation machinery upon modulating the redox status of proteins involved in translation. This crosstalk between mitochondria and protein synthesis may have an important contribution to pathologies caused by dysfunctional mitochondria.

## Abstract (web summary)

The role of reactive oxygen species (ROS) in signalling and specific targets is not fully understood. Here the authors perform a global proteomic analysis to delineate the yeast redoxome and show that increased levels of intracellular ROS caused by dysfunctional mitochondria decrease global protein synthesis.

## Introduction

Cysteine residues in proteins are involved in catalytic mechanisms, in protein stability by forming disulfide bonds or metal binding and in oxidative stress response. Cysteines often occur in vicinal dithiol structures with two other amino acids (X) in between^1^. Such CX_2_C motifs, in which the two reactive sulfhydryl groups are close to each other to form disulfides or metal clusters, represent important and well-conserved functional elements of proteins, such as oxidoreductases and iron–sulfur proteins^2^. Through reversible oxidation, cysteine residues can act as regulatory thiol-based switches in proteins providing an important post-translational control mechanism^3^. In this regard, cysteine thiols coordinating zinc ions (Zn^2+^) received considerable attention as redox switches in oxidative stress defence: the sulfur ligands can be oxidised and then reduced again with concurrent release and binding of Zn^2+^, often leading to major conformational rearrangements affecting the function of the protein^2^.

Mass spectrometry (MS) provides the potential to systematically explore the thiol redox proteome^4^. The OxICAT technology^5^, which combines differential thiol trapping and isotope-coded affinity tagging, allows for the site-specific quantification of the percentage of protein thiol oxidation in vivo. This strategy has been applied to different species, showing that cysteine residues in several proteins are partially oxidised under steady-state conditions^6^. Although in previous studies conclusions were based on a rather small number of quantified protein thiols only^5,7–10^, the potential of this MS-based approach to more comprehensively study the redoxome has recently been shown^11^. In-depth, quantitative and site-specific redox proteomic endeavours are required to gain a more comprehensive view on thiol oxidation landscapes and to uncover novel redox-active thiols that function as regulatory elements to adjust protein function to changing levels of oxidative stress. Redox-active thiols may become oxidised, reversibly or irreversibly, upon increase of reactive oxygen species (ROS) causing oxidative stress. These changes can be deleterious for protein function. However, more recently, it has been recognised that ROS can act as signalling molecules^12–15^. Thus, changes in redox-active thiols may be a part of signalling cascades, which are activated by the increase in cellular ROS. Furthermore, ROS produced by mitochondria, which constitute the major ROS production site in the cell, can serve as a means to report on mitochondrial status and activate adequate responses.

In this study, we globally analyse reversible protein thiol oxidation in the yeast *Saccharomyces cerevisiae* under basal conditions and quantify the extent of oxidation upon exogenous and intracellular mitochondria-originated oxidative stress. We site-specifically map redox-active cysteine residues as part of conserved sequence motifs in proteins of the translation machinery of the cell. We demonstrate that indeed both, exogenous and mitochondria-generated ROS, regulate the synthesis of new proteins. Our study identifies a new mechanism that involves redox switches in the translation apparatus and functions to reversibly control protein translation under conditions of increased ROS generation, including mitochondria-derived pathologies.

## Results

### Redox proteomic analysis in yeast under physiological conditions

**Fig. 1:**
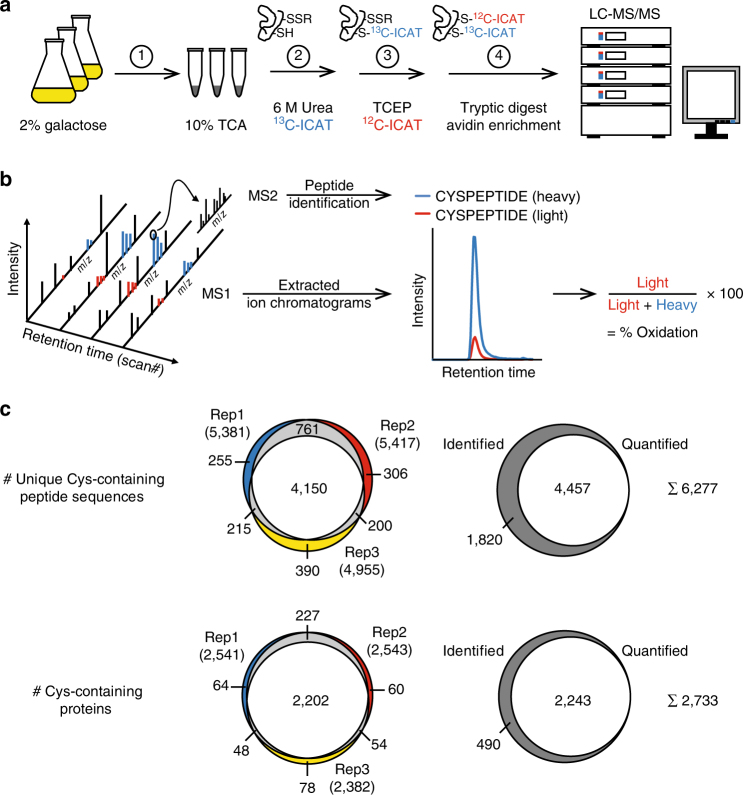
Site-resolved, large-scale analysis of the in vivo oxidation status of the yeast proteome. **a** Yeast cells grown in 2% galactose medium were immediately frozen in 10% TCA (1). Extracted proteins were denatured in 6 M urea in the presence of heavy ICAT (^13^C-ICAT) to label free thiol groups (2). Reversibly oxidised cysteine residues were reduced by TCEP and labelled by light ICAT (^12^C-ICAT) (3). Proteins were digested with trypsin and ICAT-labelled peptides were enriched by streptavidin affinity chromatography (4). Following cleavage of the biotin tag, peptides were analysed in duplicate by LC-MS/MS. **b** For the determination of the in vivo oxidation status of cysteine residues, peptides were identified based on fragment ions observed in MS2 spectra using MaxQuant (v.1.4.1.2) and then quantified by extracting MS1 ion chromatograms using Skyline (v.2.5.0). Following the integration of peak areas of heavy and light peptide variants, the proportion of reversibly oxidised cysteine residues (% oxidation) was calculated. **c** Overlap of unique cysteine-containing peptides (top) and proteins (bottom) identified in three biological replicates and the proportion of peptides/proteins quantified in least two of the three biological replicates

We performed a large-scale, quantitative and site-specific analysis of the redox status of protein cysteine residues in *S. cerevisiae*. Wild-type yeast cells were grown to mid-logarithmic growth phase on galactose-containing medium to prevent suppression of mitochondrial function. Yeast pellets were homogenised in 10% trichloroacetic acid (TCA) to retain the in vivo thiol oxidation status of proteins, followed by quantitative thiol trapping using cysteine-specific ICAT reagents^5^ and state-of-the-art liquid chromatography tandem MS (LC-MS/MS) (Fig. 1a). The data analysis included extraction of peptide ion chromatograms using Skyline^16^ to accurately determine the in vivo oxidation status of protein thiols (% oxidation) (Fig. 1b). A total of 6,277 unique cysteine-containing peptides (Cys-peptides) in 2,733 proteins were identified, of which the large majority were quantified in at least two out of the three biological replicates (Fig. 1c and Supplementary Data 1). The ICAT-based approach proved to be highly effective as demonstrated by the high number of identified unique cysteine-containing peptide sequences compared with shut-gun proteomic data of the whole yeast proteome^17,18^ (Supplementary Fig. 1a). The analysis covered the whole abundance range of the yeast proteome with only a slight bias toward more abundant proteins (Supplementary Fig. 1b). Our data therefore provide a large resource for the in vivo oxidation status of protein thiols, which significantly expands previous OxICAT data of different eukaryotic systems (Supplementary Fig. 1c). The measured protein redox profiles were highly reproducible with average Pearson’s correlation coefficients of 0.96 for biological replicates and 0.95 for technical replicates (Supplementary Fig. 1d and e).

**Fig. 2:**
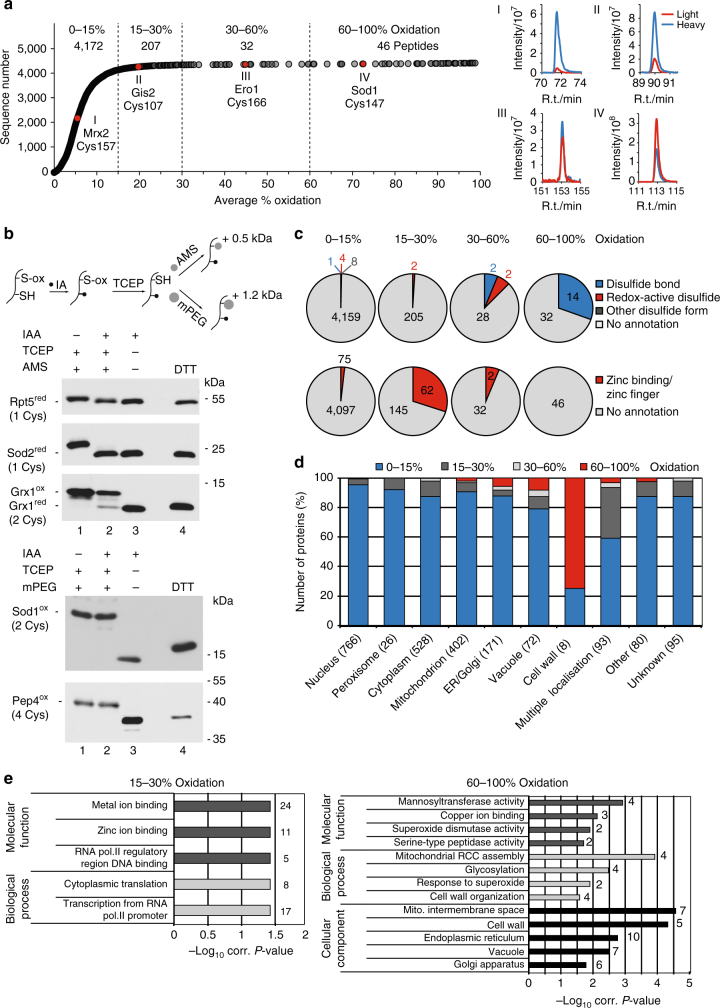
The basal yeast thiol oxidation landscape. **a** Distribution of the in vivo oxidation status of 4,457 unique cysteine-containing peptides. The dataset was classified into four oxidation groups as indicated. For each group, a representative peptide (red) and the respective extracted ion chromatogram (XIC; right) are shown. XICs display the redox state of (I) Cys157 of the methionine-R-sulfoxide reductase Mxr2, (II) Cys107 of the translational mRNA activator Gis2, (III) Cys166 of the thiol oxidase Ero1 and (IV) Cys147 of the cytosolic superoxide dismutase Sod1. Red line, light peptide peak (oxidised); blue line, heavy peptide peak (reduced). **b** Top: schematic illustration of the thiol-trapping approach. The reduced cysteine residue of a protein is alkylated by iodoacetamide (IAA). The oxidised cysteine residue is reduced by TCEP and is thus accessible for the thiol-modifying agent AMS or mPEG. Modification results in a shift of 0.5 or 1.2 kDa, respectively, per modified cysteine residue. Bottom: when indicated, proteins of total yeast cell extracts were incubated with 10 mM IAA, 50 mM TCEP and 10 mM AMS or 10 mM mPEG. As a control, one sample was incubated with 100 mM DTT. Proteins were separated by SDS-PAGE followed by immunodecoration with specific antibodies. **c** Frequency of disulfide bond (top) and zinc binding and/or zinc finger annotations (bottom) within different oxidation groups of cysteine-containing peptides as indicated. Other disulfide forms include transient disulfide bonds and those in linked or nuclear-retained form. **d** Distribution of proteins of different oxidation groups according to subcellular location. Gene ontology (GO) slim terms for cellular components were retrieved from the *Saccharomyces* genome database with the number of proteins in parenthesis. Proteins were grouped according to the most oxidised peptide. **e** GO term enrichment analysis of proteins with peptides in the oxidation range 15–30% (left) and 60–100% (right). For each term, the number of proteins is shown

We classified all quantified peptides according to their average oxidation levels in four groups: 0–15%, 15–30%, 30–60% and 60–100% oxidation (Fig. 2a). In wild-type cells, the vast majority of protein thiol groups were reduced (0–15% oxidation, 4,172 Cys-peptides) and only a small set of proteins contained oxidised cysteine residues (Supplementary Data 2). In fact, we quantified few peptides (46, 1%) with highly oxidised thiol groups including, for example, Cys147 of superoxide dismutase 1 (Sod1) (Fig. 2a, inset IV) involved in intramolecular disulfide bond formation, which is essential for Sod1 activity^19^. To examine the redox status of organelle proteins, the organelles are frequently isolated from the entire cell prior to analysis. We compared the oxidation status of Cys-peptides from whole cell extracts and isolated mitochondria (Supplementary Fig. 1f-i and Supplementary Data 3). The overall oxidation level of Cys-peptides increased considerably through mitochondria isolation. Thus, the data on the thiol oxidation status of organellar proteins obtained from isolated organelles should be taken with caution since the isolation procedure may alter the in vivo redox status of cysteine residues.

To validate our in vivo thiol oxidation data from whole yeast cells, we investigated the redox status of selected proteins by applying a thiol-trapping approach to whole cell protein extracts (Fig. 2b, schema). The proteins Rpt5 and Sod2 contain one cysteine residue each and these were oxidised < 10% according to our MS data (Supplementary Data 2). In line with this, we observed both proteins in the reduced state in our thiol-trapping approach. Using MS, we found that a fraction of Grx1, which comprises two cysteine residues, was present in the oxidised state (Supplementary Data 2). Indeed, our shift assay revealed two pools of Grx1 in the cell, an oxidised and a reduced one, in line with our MS data. We further determined an average oxidation level of 70% for Sod1 (two cysteines) and 90% for Pep4 (four cysteines, three of which were quantified) (Supplementary Data 2). Indeed, we also observed both proteins in the oxidised state in our biochemical approach (Fig. 2b). Thus, our biochemical data are in agreement with the results of our MS-based study.

Next, we performed a functional analysis of the peptide and protein groups based on their thiol oxidation levels. Information about cysteine residues with annotated disulfide bonds (Fig. 2c, upper panel) or zinc-binding properties (Fig. 2c, lower panel) were retrieved from the UniProt knowledge database. As expected, the group of highly oxidised thiols (60–100% oxidation) was enriched for cysteines known to be involved in disulfide bond formation. In this group were additional 32 thiol-containing peptides with no available annotation and these may also contribute to the formation of structural disulfide bonds in proteins. Zinc-binding cysteine residues were found predominantly in the group of 15–30% oxidation (Fig. 2c). We examined the cellular localisation of the proteins with Cys-peptides within the different ranges of oxidation. Proteins with highly oxidised thiol groups were localised to mitochondria, the endoplasmic reticulum/Golgi apparatus, the vacuole and the cell wall (Fig. 2d). Gene ontology (GO) enrichment analysis confirmed an overrepresentation of these cellular localisations in the range of 60–100% oxidation (Fig. 2e, right panel). Interestingly, proteins with potentially redox-sensitive thiols (15–30% oxidation) contained several metal ion-binding proteins (see also Fig. 2c) and proteins with a function in cytoplasmic translation and transcription (Fig. 2e, left panel).

In summary, the vast majority (93.6%) of proteins exists in the reduced state and the group of potentially redox-sensitive proteins is enriched for factors with zinc-binding motifs and/or with a role in transcriptional and translational processes.

### Changes in thiol oxidation upon oxidative stress

**Fig. 3:**
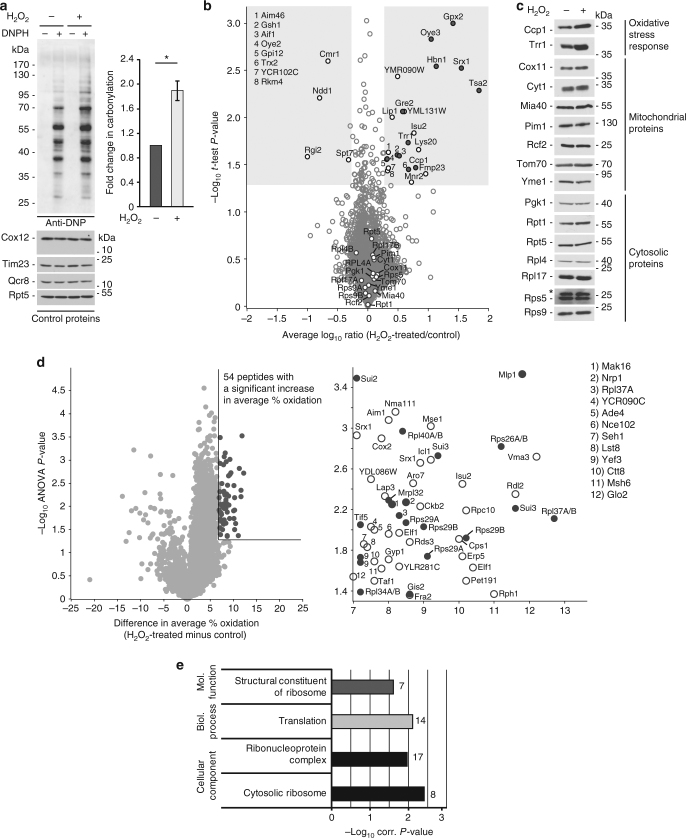
Hydrogen peroxide treatment of wild-type yeast cells. **a** Carbonylation of proteins in wild-type cells treated with 1 mM H_2_O_2_ for 30 min was analysed by immunoblotting with anti-DNP antibody or quantified by spectrophotometry. Mean ± SEM, *n* = 3, *P-value < 0.05; two-sided, paired *t*-test DNP(H), 2,4-dinitrophenyl(hydrazine). **b** Volcano plot showing H_2_O_2_-dependent changes in protein abundance. In total, 3,116 unique proteins and 61 protein groups were quantified. Shaded areas highlight proteins with an average fold change of ≥ 2 (average log_10_ ratio = ± 0.3) and a *P-*value < 0.05 (−log_10_*P-*value > 1.3; *n* = 3, two-sided *t*-test). Dark grey, regulated proteins with the GO term ‘response to oxidative stress’ and/or ‘oxidoreductase activity’. **c** Wild-type cells were grown in fermentative medium at 28 °C and when indicated treated with 1 mM H_2_O_2_ for 30 min. Samples were analysed by immunoblotting. *Unspecific band. **d** Left: volcano plot showing difference in average % oxidation (H_2_O_2_-treated minus untreated samples) for 4,341 unique cysteine-containing peptides plotted against −log_10_*P-*value highlighting unique cysteine-containing peptides with significantly increased oxidation levels (dark grey, *P-*value < 0.05, *n* = 3, ANOVA). Lines indicate difference in average % oxidation > 7 and a *P-*value of 0.05 (−log_10_ = 1.3). Right: zoom-in of the region highlighted in the volcano plot. Peptides of proteins annotated with the GO term ‘ribonucleoprotein complex’ are highlighted (dark grey). **e** GO term enrichment analysis of proteins with significantly increased oxidation levels. *P-*values after Benjamini–Hochberg FDR (< 0.05) correction were plotted against their corresponding GO terms from the three main domains (cellular component, molecular function and biological process). For each GO term, the number of proteins is shown

To reveal reversible changes in the thiol redox proteome upon oxidative stress, we induced oxidative stress in wild-type yeast using hydrogen peroxide (H_2_O_2_). Applying 1 mM of H_2_O_2_ for 30 min to logarithmically growing yeast cells was sufficient to detect an increase in both ROS (Supplementary Fig. 2a) and protein carbonylation (Fig. 3a), the latter indicating the irreversible oxidation of proteins by ROS. These were benchmarks for our MS-based study to confirm that the oxidative stress we applied had an effect on proteins. We first examined whether our experimental conditions of H_2_O_2_ treatment led to alterations in protein abundance using stable isotope dimethyl labelling^20^ (Fig. 3b and Supplementary Data 4). The quantitative proteome data (*n* = 3) were confirmed by immunoblot analysis of selected proteins (Fig. 3c). Proteins involved in oxidative stress defence (e.g., Ccp1 and Trr1) were significantly upregulated, whereas the levels of mitochondrial, ribosomal and other cytosolic proteins remained largely unaffected (Figs. 3b, c and Supplementary Data 4).

We analysed the in vivo reversible oxidation landscape of the H_2_O_2_-treated yeast cells following the same experimental approach as described for the basal redoxome (see Fig. 1a). We identified 4,833 and quantified 4,362 (89.9%) Cys-peptides, corresponding to 2,354 identified and 2,225 (94.5%) quantified proteins (Supplementary Fig. 2b). Both biological and technical reproducibility of our global redox proteomic analysis were high (Supplementary Fig. 2c), allowing for a quantitative assessment of the in vivo oxidation status of 4,341 unique Cys-peptides of 2,214 proteins in H_2_O_2_-treated vs. untreated yeast cells (Supplementary Fig. 2b and Supplementary Data 5). As a consequence of oxidative stress, most protein thiols gained oxidation, but the increase in average oxidation did not exceed 13% (Supplementary Fig. 2d and e). We analysed the redox status of Grx1 following treatment of yeast cells with H_2_O_2_ by immunoblotting. However, due to the limited sensitivity of the immunoblot analysis, a substantial increase in the oxidised pool of Grx1 protein was detected only when cells were exposed to higher concentration than 1 mM of H_2_O_2_ (Supplementary Fig. 2f, left part). This increase in oxidation of the Grx1 protein was generally in agreement with our redox proteome data, showing already a slight increase (6.9%) in peptide thiol oxidation at 1 mM H_2_O_2_ (Supplementary Fig. 2f, right part; Supplementary Data 5).

We determined a set of 54 unique Cys-peptides comprising 67 cysteine residues in 47 proteins with an increase in oxidation of ≥ 7% and a *P-*value < 0.05 (*n* = 3) (Fig. 3d and Supplementary Data 6). Notably, various constituents of the small and large subunit of cytoplasmic ribosomes, but also other factors involved in translation were identified to be sensitive to H_2_O_2_ (Fig. 3d, right inset, Supplementary Data 6). Next, we identified functionally related proteins within the group of H_2_O_2_-sensitive proteins by GO enrichment analysis. We compared all proteins with peptides exhibiting increased thiol oxidation of ≥ 7% (*P-*value < 0.05, *n* = 3) (Fig. 3d and Supplementary Data 6) to all proteins with Cys-peptides quantified in the control (Supplementary Data 2). Ribosomal proteins, proteins involved in ribosome biogenesis and in cytosolic translation were overrepresented in our analysis (Fig. 3e). The presence of a second thiol in close proximity is a strong predictor for the oxidation susceptibility of a cysteine residue^21^. Indeed, when analysing the frequency distribution of amino acids within H_2_O_2_-sensitive sequences, we observed vicinal cysteines in a CX_2_C constellation in 23 out of 54 (42.6%) cases (Supplementary Fig. 3a). Closer inspection of the redox-sensitive thiols in proteins involved in cytosolic translation revealed that virtually all contain CX_2_C-X_(9–47)_-CX_(2,4)_C sequence motifs (Table 1). Interestingly, one protein of this group was a mitochondrial ribosomal protein, Mrpl32, which was shown earlier to undergo redox-dependent regulation^22^. We studied published structures of proteins that are active in translation and contain this motif. In six out of seven cases, we found the four cysteines within the motif to assemble in a tetrahedral geometry coordinating a central Zn^2+^ ion (Supplementary Fig. 3b). These proteins contain distinct structural elements, i.e., an N-terminal β-hairpin and a C-terminal α-helix, which provide two zinc ligands each, which are characteristic for a class of structural zinc fingers termed treble clefs^23^. Here, the binding of Zn^2+^ allows the spatial coordination of more distant cysteine residues and stabilises the folding of the protein domains. Multiple sequence alignments show that the cysteines within these sequences are highly conserved throughout evolution (Supplementary Fig. 3c). We conclude that translational apparatus components with their conserved CX_2_C-X_(9–47)_-CX_(2,4)_C motifs coordinating Zn^2+^ are targets of ROS-dependent regulation.

**Table 1:** Sequence motif in H_2_O_2_-sensitive proteins

Sequence motif	Amino acid residues	Gene name	Protein name
CX_2_C-X_47_-**C**X_2_**C**	23–77	RPS26A/RPS26B	40S ribosomal protein S26-A/S26-B
CX_2_**C**-X_14_-**C**X_2_C	21–42	RPS29A/RPS29B	40S ribosomal protein S29-A/S29-B
**C**X_2_**C**-X_33_-CX_2_C	44–84	RPL34A/RPL34B	60S ribosomal protein L34-A/L34-B
**C**X_2_C-X_11_-**C**X_2_**C**	19–37	RPL37A/RPL37B	60S ribosomal protein L37-A/L37-B
CX_2_C-X_10_-CX_4_**C**	96–115	RPL40A/RPL40B	Ubiquitin-60S ribosomal protein L40
**C**X_2_**C**-X_9_-CX_2_C	104–119	MRPL32	54S ribosomal protein L32, mitochondrial
**C**X_2_C-X_19_-**C**X_2_C	236–262	SUI3	Eukaryotic translation initiation factor 2 SU beta
**C**X_2_**C**-X_18_-CX_2_C	99–124	TIF5	Eukaryotic translation initiation factor 5

Proteins involved in translation comprise H_2_O_2_-sensitive thiols (*P-*value < 0.05, *n* = 3; difference in % oxidation ≥ 7) within CX_2_C-X_(9–47)_-CX_(2,4)_C motifs. H_2_O_2_-sensitive cysteine residues are highlighted in bold

### Mitochondrial ROS production causes protein thiol oxidation

**Fig. 4:**
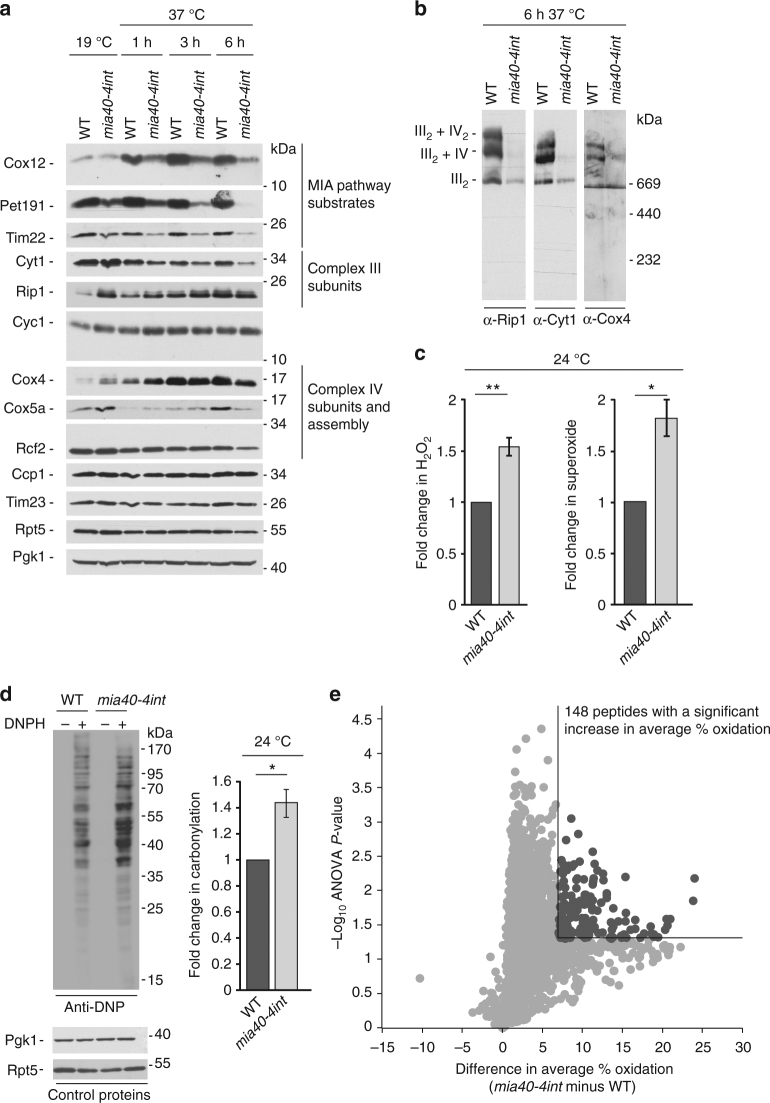
MIA mutant shows increased levels of ROS. **a** Wild-type yeast and *mia40-4int* mutant were grown in respiratory medium at 19 °C and shifted to 37 °C. Samples were collected at the indicated time points and analysed by immunoblotting. **b** Mitochondria were isolated from wild-type cells and *mia40-4int* mutant and analysed by Blue Native gel electrophoresis followed by immunoblotting. **c** Endogenous levels of H_2_O_2_ and superoxide of wild-type cells and *mia40-4int*. Mean ± SEM, *n* = 3. *P-value < 0.04; **P-value < 0.02; two-sided, paired *t*-test. **d** Carbonylation of proteins in wild-type cells and *mia40-4int* mutant was analysed by immunoblotting with anti-DNP antibody or quantified by spectrophotometry. Mean ± SEM, *n* = 3. *P-value < 0.05; two-sided, paired *t*-test. DNPH, 2,4-dinitrophenylhydrazine. **e** Volcano plot showing difference in average % oxidation (*mia40-4int* minus wild-type) for 2,099 unique cysteine-containing peptides plotted against the −log_10_*P-*value (ANOVA test, *n* = 4). Cysteine-containing peptides with significantly increased oxidation levels are highlighted in dark grey (% oxidation ≥ 7, *P-*value < 0.05, *n* = 4). Lines indicate a difference in average % oxidation of ≥ 7 and a *P-*value of 0.05 (−log_10_ = 1.3). WT, wild-type

To investigate whether changes in protein thiol oxidation upon H_2_O_2_ are physiologically relevant, we aimed at analysing an endogenous situation of elevated ROS production. Mitochondria are a major source of cellular ROS and increased levels of ROS can occur as a result of increased electron leakage of dysfunctional respiratory chain complexes. The Mia40 mitochondrial protein translocase is responsible for the import of intermembrane space proteins^24^. Mia40 has a critical role in the biogenesis of its substrate proteins including components and assembly factors of respiratory chain complexes^24–26^. Mia40 is essential in yeast. Thus, to analyse dysfunctional Mia40, we used yeast expressing a mutant version, *mia40-4int*, which exhibits a thermo-sensitive phenotype. Under permissive conditions, the abundance of some proteins of the respiratory chain complexes was decreased, whereas other proteins remained unchanged or were increased (Fig. 4a). With escalating temperature restriction, the imbalance in respiratory chain complexes was transformed into a massive decrease in many respiratory chain subunits in *mia40-4int* (Fig. 4a). Upon a 6 h shift to higher temperature, respiratory chain complexes declined in *mia40-4int* (Fig. 4b). The observed imbalance in the respiratory chain complexes in *mia40-4int* could be a reason for an increase in ROS production. Indeed, we observed a boost in ROS production in the *mia40-4int* mutant (Fig. 4c and Supplementary Fig. 4a). This finding was supported by an increase in protein carbonylation as a consequence of elevated ROS levels (Fig. 4d and Supplementary Fig. 4b). Moreover, we found that the elevation in ROS production and protein carbonylation were also an attribute of other conditional *mia40* mutants (Supplementary Fig. 4c and d). We applied the OxICAT strategy to the *mia40-4int* mutant (see Fig. 1a, b). Both *mia40-4int* and wild-type yeast cells were grown in respiratory medium and shifted to restrictive temperature (37 °C) for 3 h. LC-MS analysis resulted in the quantification of 2,099 Cys-peptides in 1,300 proteins in at least three out of four biological replicates of both wild-type and mutant cells (Supplementary Data 7). We found that most cysteine residues were more oxidised in the *mia40-4int* mutant compared with wild-type yeast cells (Supplementary Fig. 4e and f and Supplementary Data 7). A total of 148 Cys-peptides showed a significant increase in oxidation (≥ 7%, *P-*value < 0.05, *n* = 4) (Fig. 4e and Supplementary Data 7). We analysed the occurrence of disulfide bonds and zinc-binding properties in the wild-type control grown on respiratory medium (37 °C, 3 h). The analysis revealed a similar distribution within different groups of oxidation as for wild-type yeast cells grown on fermentative medium at 28 °C (Supplementary Fig. 4g; see Fig. 2c).

**Fig. 5:**
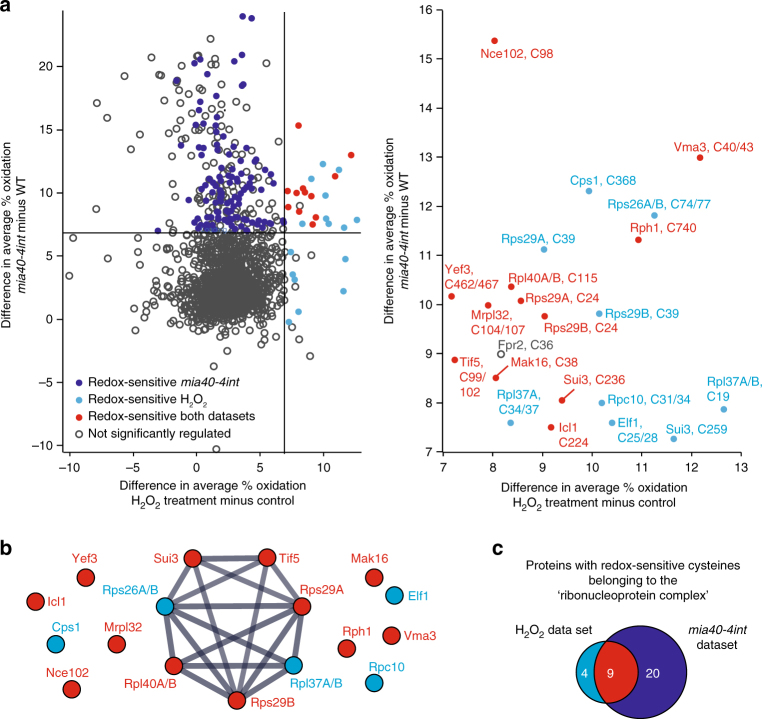
Comparison between H_2_O_2_-induced and *mia40-4int*-dependent changes in thiol oxidation **a** Left: comparison of difference in average % oxidation values for 1,785 unique cysteine-containing peptides quantified in both datasets. Lines indicate difference in average % oxidation ≥ 7. Right: zoom-in of the region with an average increase in oxidation of at least + 7% in *mia40-4int* and upon H_2_O_2_ treatment. Peptides are labelled with the respective protein names and the position(s) of cysteine residue(s) in the sequence. Unique cysteine-containing peptides that show an increase in thiol oxidation of at least + 7% with a *P-*value < 0.05 in *mia40-4int* (*n* = 4) and/or upon H_2_O_2_ treatment (*n* = 3) are highlighted as indicated. Cysteine-containing peptides highlighted in blue in the zoom-in also exhibited increased thiol oxidation in *mia40-4int* (≥ 7%) but with *P-*values > 0.05 (*n* = 4). **b** Functional protein association network of proteins which exhibited increased oxidation (≥ 7%) in *mia40-4int* and in WT yeast upon H_2_O_2_ treatment using STRING^63^ (version 10.0). Proteins highlighted in red showed a significant increase in thiol oxidation in *mia40-4int* (*P-*value < 0.05, *n* = 4), as well as upon H_2_O_2_ treatment (*P-*value < 0.05, *n* = 3). Proteins highlighted in blue exhibited a significant increase in thiol oxidation upon H_2_O_2_ treatment (≥ 7%, *P-*value < 0.05, *n* = 3). Thiol oxidation of these proteins was also increased in *mia40-4int* (≥ 7%) with *P-*values > 0.05 (*n* = 4). **c** Comparison of the number of proteins annotated with the GO term ‘ribonucleoprotein complex’ with at least one unique cysteine-containing peptide with an increase in thiol oxidation of at least + 7% (*P-*value < 0.05) upon H_2_O_2_ treatment (*n* = 3) and/or in *mia40-4int* (*n* = 4)

Next, we compared the protein thiol oxidation status of H_2_O_2_-treated cells with the data obtained for *mia40-4int*-dependent changes. A total of 1,785 unique Cys-peptides were reproducibly quantified in both datasets. The respective cysteine-containing peptides in the corresponding control samples were mostly reduced (Supplementary Fig. 4h left) and shifted to higher oxidation upon stress conditions (Supplementary Fig. 4h right). Within the overlapping quantified peptides, *mia40-4int* showed a higher increase in average % oxidation compared to H_2_O_2_-treated cells (Supplementary Fig. 4h right), whereas the redox state in the corresponding control conditions was comparable (Supplementary Fig. 4h left). We identified 12 peptides comprising 16 cysteine residues that were significantly more oxidised (≥ 7% oxidation, *P-*value < 0.05) in both datasets (Fig. 5a, proteins highlighted in red). An additional 9 peptides comprising 13 cysteine residues also showed increased oxidation (≥ 7% oxidation) in both datasets but with a *P-*value < 0.05 in the H_2_O_2_ dataset only (Fig. 5a, proteins highlighted in blue). The respective proteins were functionally related and were predominantly part of the machineries regulating the synthesis of proteins (Fig. 5b). Further analysis of the *mia40-4int* data in respect to proteins that belong to ‘ribonuleoprotein complex’ revealed additional proteins with redox-sensitive cysteine residues that were quantified and not found to be regulated upon H_2_O_2_ stress (Fig. 5c and Supplementary Data 7). These included factors important for the biogenesis of ribosomes (Tsr1 and Cms1) and ribosomal RNAs (Rrp12 and Rrp5) or the nuclear export of ribosome subunits (Crm1).

In summary, ROS originating from mitochondria causes changes in the in vivo redox status of proteins including several factors involved in the regulation of protein synthesis. These findings are partially overlapping but also exceeding the redox changes observed upon H_2_O_2_ treatment.

### Mitochondrial ROS are linked to cytosolic translation attenuation

**Fig. 6:**
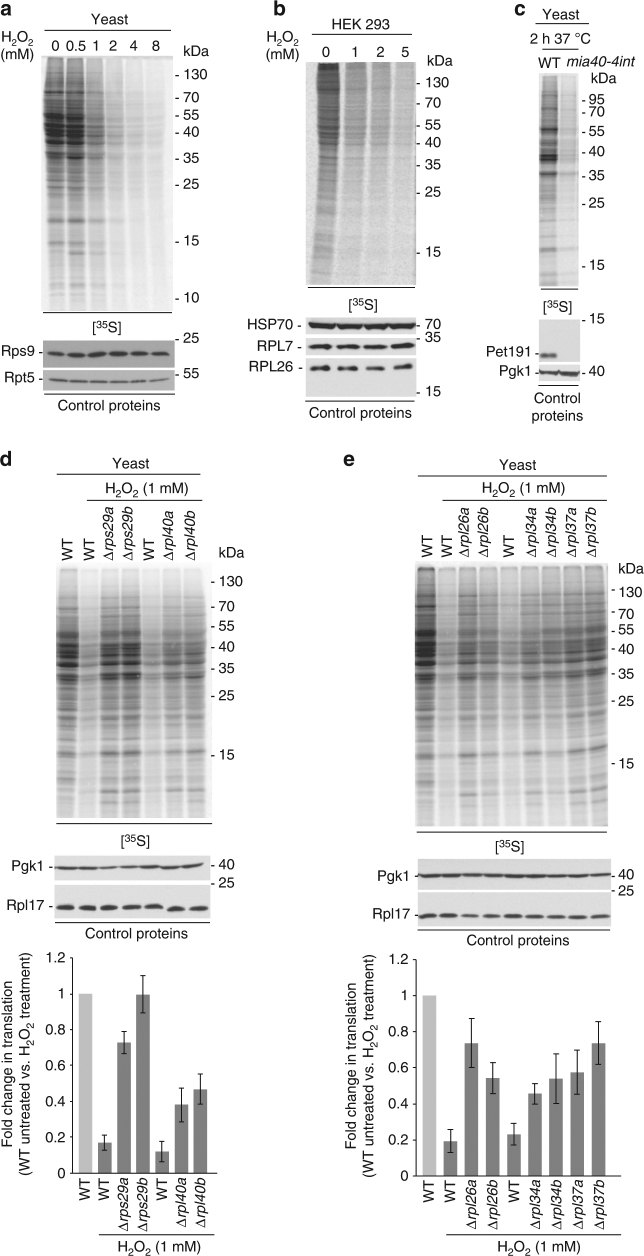
ROS induction reduces protein translation in the cytosol. **a**–**e** Incorporation of [^35^S]-labelled amino acids in yeast or mammalian cells. Total cell extracts were separated by SDS-PAGE and analysed by autoradiography or immunodecorated with specific antibodies. **a** Wild-type (BY4741) yeast cells were grown on fermentative medium and treated for 30 min with H_2_O_2_ as indicated. **b** HEK 293 cells were treated for 2 h with H_2_O_2_ as indicated. **c** Yeast cells were grown on respiratory medium at 19 °C and shifted for 2 h to restrictive temperature (37 °C). Incorporation of [^35^S]-labelled amino acids in wild-type yeast and *mia40-4int* mutant was done for 1 h before collection of cells. **d**, **e** Wild-type (BY4741) yeast cells and cells with deletions of ribosomal genes (encoding for proteins that according to the proteomics data were significantly more oxidised in *mia40-4int* and upon H_2_O_2_ treatment (**d**) or significantly more oxidised upon H_2_O_2_ treatment only (**e**)) were grown on fermentative medium and treated for 30 min with H_2_O_2_ (upper panel). Quantification of protein synthesis. Mean ± SEM, *n* = 3 (lower panel). WT, wild-type

**Fig. 7:**
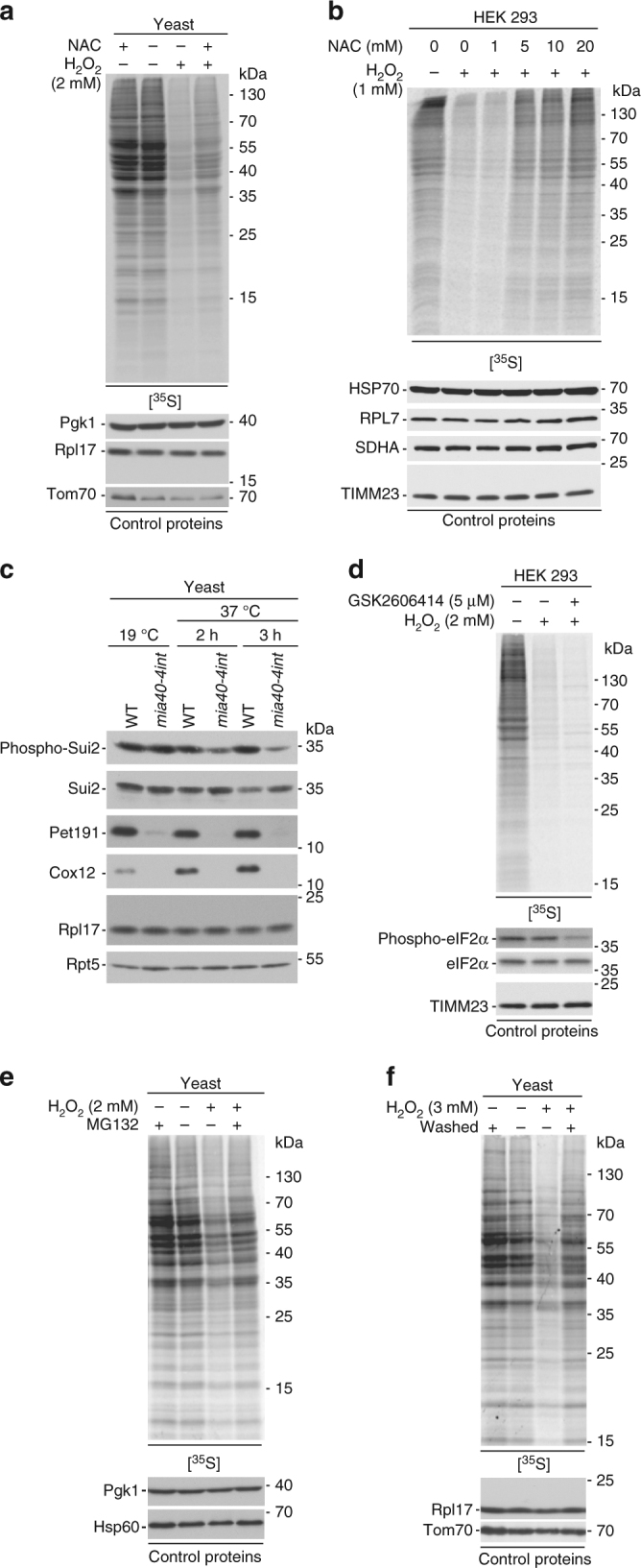
Translation initiation block is not responsible for inhibitory effect of ROS. **a**, **b**, **d**–**f** Incorporation of [^35^S]-labelled amino acids in yeast or mammalian cells. Total cell extracts were separated by SDS-PAGE and analysed by autoradiography or immunodecorated with specific antibodies. **a** Wild-type (BY4741) yeast cells were grown on fermentative medium supplemented with 100 mM *N*-acetylcysteine (NAC) for 4 h. During the last 30 min of culturing, H_2_O_2_ was added. **b** HEK 293 cells were treated for 2 h with H_2_O_2_ in the presence of NAC as indicated. **c** Yeast cells were grown on respiratory medium at 19 °C and shifted for 2 or 3 h to restrictive temperature (37 °C). Samples were analysed by immunoblotting using specific antibodies. **d** HEK 293 cells were treated for 3 h with the PERK kinase inhibitor GSK2606414. H_2_O_2_ was added after 1 h of treatment with GSK2606414. **e** Wild-type (YPH499) yeast cells were treated for 3 h with 25 µM MG132. H_2_O_2_ was added 30 min prior harvesting of cells. **f** Wild-type (YPH499) yeast cells were treated with H_2_O_2_ for 30 min. Cells were washed and further incubated for 1 h. Incorporation of [^35^S]-labelled amino acids was done at the same time as treatment with H_2_O_2_ or for 1 h starting after washing. WT, wild-type

Our redox proteomic results revealed that oxidative stress induced by H_2_O_2_ treatment modulates the oxidation of ribosomal subunits and further proteins involved in translation (Supplementary Fig. 3b and c and Table 1). We treated wild-type yeast with different concentrations of H_2_O_2_ and analysed the incorporation of radiolabelled amino acids into newly synthesised proteins. Global translation was reduced with increased concentration of H_2_O_2_ in two different wild-type yeast strains (Fig. 6a and Supplementary Fig. 5a). Reduction of protein synthesis was not caused by massive cell death (Supplementary Fig. 5b and c). We asked whether the effect of oxidative stress on translation in yeast is also present in mammalian cells. The treatment of HEK 293 cells with H_2_O_2_ decreased translation in a concentration-dependent manner (Fig. 6b). The abundance of the ribosomal proteins examined (i.e., RPL7 and RPL26) was not changed (Figs. 6b and 7b). Cell death, after the treatment with H_2_O_2_, was minor and thus could not account for the decrease in overall protein synthesis (Supplementary Fig. 5d).

Recently, others and we discovered that cytosolic translation is modulated in cells harbouring defective mitochondria^27,28^. Translational inhibition was observed in several mutants defective in protein import into mitochondria, including the ROS-producing conditional mutants of Mia40 (Fig. 6c and Supplementary Fig. 5e). These experiments demonstrate a link between ROS produced by defective mitochondria and effects on the translational apparatus resulting in the inhibition of protein synthesis.

Next, we analysed protein synthesis in the deletion mutants of the ribosomal proteins (Supplementary Data 6 and Supplementary Data 7) that were more oxidised in *mia40-4int*-dependent manner and/or upon H_2_O_2_ treatment (Figs. 6d, e and Supplementary Fig. 5f and g). As many ribosomal proteins in yeast, they are encoded by two paralogues genes (e.g., Rps29a and Rps29b). Thus, deletion of only one gene was not lethal and did not significantly alter protein synthesis under physiological conditions (Supplementary Fig. 5f and g). However, the response to attenuate translation upon H_2_O_2_ treatment was decreased in the cells lacking these ribosomal proteins (Figs. 6d, e). Further, we constitutively expressed cysteine mutant forms of *RPL40A*-C115 (C115 was found to be sensitive to oxidative stress in our redoxome analyses; Fig. 5, Supplementary Fig. 3b and Supplementary Data 6 and 7) in wild-type yeast cells and analysed changes in translation (Supplementary Fig. 5h). Cells expressing the oxidation mimicking *RPL40A*-C115D version and empty vector control were equally sensitive to H_2_O_2_ treatment (Supplementary Fig. 5h). The translation inhibition response was less pronounced for the cells with the *RPL40A*-C115S form (Supplementary Fig. 1h). Altogether, these findings supported our MS-based data, suggesting that ribosomal proteins can act as sensors for oxidative stress in order to mediate a decrease in translation.

### Oxidative stress causes cytosolic translation inhibition

To address the issue whether there is a causal dependence between ROS and translation inhibition, we investigated whether ROS are sufficient to cause a decrease in protein synthesis. If ROS are a signal to decrease global protein synthesis, the reduction of ROS levels should restore protein synthesis. We used the well-characterised compound *N*-acetyl-l-cysteine (NAC), an antioxidant that scavenges ROS and a chemical precursor of glutathione^29^, to investigate the inhibitory effect on cytosolic protein synthesis (see Figs. 7a, b and Supplementary Fig. 6a, c, d). Indeed, the reduction in protein synthesis could be in part reversed in wild-type yeast by applying NAC in combination with H_2_O_2_ (Fig. 7a), but also when cells were pretreated with NAC prior H_2_O_2_ exposure (Supplementary Fig. 6a). NAC treatment alone did not affect protein synthesis (Fig. 7a and Supplementary Fig. 6a). Moreover, ebselen, a compound acting as a mimic of glutathione peroxidase^30,31^, partially restored translation upon H_2_O_2_ treatment in wild-type yeast (Supplementary Fig. 6b). Further, we applied NAC to mammalian cells to rescue the translation defect upon oxidative stress. NAC treatment in various concentrations had no impact on protein synthesis itself (Supplementary Fig. 6c). However, NAC was able to counteract the translational defects observed after H_2_O_2_ (Fig. 7b). When the *mia40-4int* cells were treated with the antioxidant NAC, translation was partially restored (Supplementary Fig. 6d). These results are consistent with the hypothesis that the production of ROS by defective mitochondria is a cause of translation inhibition.

### ROS affect post-initiation steps of translation

A decrease in global translation is a general response of the cell to various stress conditions^32–39^. Translation initiation is often inhibited via the phosphorylation of the eukaryotic initiation factor 2α (eIF2α)^32^. We analysed the dynamics of the phosphorylation of eIF2α (yeast Sui2) following treatment of yeast cells with increasing concentrations of H_2_O_2_ in yeast. Phosphorylation of eIF2α increased upon low concentrations of H_2_O_2_ but decreased when the oxidative stress became more acute (Supplementary Fig. 7a), which is consistent with previous observations^40^. However, translation declined steadily with increasing H_2_O_2_ concentrations (see Fig. 6a). This showed that inhibition of eIF2α via phosphorylation did not fully correlate with the observed decrease in translation. Most importantly, phosphorylation of eIF2α was decreased in the *mia40-4int* mutant under restrictive temperature (37 °C) compared with wild-type cells (Fig. 7c), and thus this phosphorylation-dependent mechanism cannot contribute to the translation attenuation. In line with the findings in yeast cells, we did not observe an increase in eIF2α phosphorylation in HEK 293 cells upon H_2_O_2_ treatment (Fig. 7d). Furthermore, reduction of eIF2α phosphorylation by inhibition of PERK (PKR-like endoplasmic reticulum kinase)^41^, a kinase phosphorylating eIF2α^42^, did not reverse the translation defect (Fig. 7d).

Inhibition of mammalian target of rapamycin (mTOR) pathway was shown to regulate translation via multiple ways including inhibition of eukaryotic translation initiation factor 4 (eIF4E)^43^. A native protein inhibitor, eIF4E-binding protein 1 (4E-BP1), when phosphorylated, releases eIF4E for translation initiation. We analysed phosphorylation of 4E-BP1 in HEK 293 cells. Upon H_2_O_2_ treatment, 4E-BP1 was less phosphorylated resulting in stronger binding of eIF4E and inhibition of translation, indicative for mTOR pathway inhibition (Supplementary Fig. 7b). Inhibition of mTOR by use of the inhibitor INK128^44^ resulted in a decrease in translation. However, simultaneous H_2_O_2_ treatment had an additive effect on translation attenuation (Supplementary Fig. 7c), suggesting the involvement of additional mTOR-independent mechanisms mediated by oxidative stress. In yeast, mutants with deletions of *TOR1* (component of TOR complex 1), *SCH9* (homologue of S6 kinase) or *TIF3* (homologue of eIF4B and target of Sch9)^45^ showed higher translation compared to wild-type control upon H_2_O_2_ treatment. However, translation was still drastically reduced compared to untreated samples (Supplementary Fig. 7d), consistently pointing to an additional TOR-independent mechanism. As mTOR pathway inhibition via a block in translation initiation was not the sole cause of translation inhibition upon oxidative stress, we investigated whether later stages of translation are affected. If the elongation or termination of translation are blocked, nascent chains, which are removed by the ribosome quality control mechanisms and degraded^46,47^, should be recovered upon proteasomal inhibition. Indeed, treatment of yeast cells with the proteasome inhibitor MG132 prior H_2_O_2_ treatment led to the partial rescue of newly synthesised proteins (Fig. 7e). Our data suggest a mechanism involved in translation inhibition upon oxidative stress, which is distinct from TOR regulation and acts via inhibition of translation elongation or termination.

### Translation attenuation upon ROS action is a reversible process

Scavenging ROS by antioxidants to restore translation did not fully exclude that the translation machinery components were simply damaged, resulting in the observed translation block. Thus, we attempted to investigate restoration of the translation defect by removing the source of stress. Yeast cells were first treated with H_2_O_2_, then washed to remove H_2_O_2_, and afterwards the production of newly synthesised proteins was monitored. Indeed, translation was fully restored in these cells (Fig. 7f). Along the same line, translation inhibition in the temperature-sensitive *mia40-4int* mutant could be restored to wild-type levels by shifting the mutant cells back to permissive temperature, the condition under which the *mia40-4int* mutant did not exhibit a strong phenotype (Supplementary Fig. 7e). These findings show that reversible changes underlie translation attenuation.

In summary, oxidative stress imposed externally or caused by mitochondrial pathologies within the cell is sufficient to influence protein synthesis. The process of translation remodelling can be mediated by reversible changes in the redox state of components involved in protein synthesis that are susceptible to oxidation (Supplementary Fig. 7f).

## Discussion

In this study, we provide a rich resource of the in vivo thiol oxidation status of approximately 4,300 unique Cys-peptides in more than 2,200 yeast proteins under basal growth conditions, which expands the current data on the thiol proteome of *S. cerevisiae*^7^ by more than 10-fold. We found that the large majority of cysteine residues are virtually fully reduced under physiological conditions with an average degree of oxidation of 7.4%, which is generally in line with previous studies performed in different organisms ranging from yeast to human cells^7–11^. We found that exposure of yeast to a non-toxic dose of H_2_O_2_ led to an overall increase in the in vivo oxidation status of the thiol proteome. However, changes in protein thiol oxidation were not greater than plus 13% on average. Interestingly, steady state levels of proteins involved in oxidative stress response were considerably increased, indicating that the cells immediately respond to H_2_O_2_ by activating a specific, ROS-induced gene transcriptional programme^15,48^. Other studies also reported only minor changes in the global in vivo thiol oxidation status of proteins following exposure to H_2_O_2_ or other ROS-generating stresses^10,11^, suggesting that ROS execute a specific rather than a global function on protein thiols.

Our study identified redox-sensitive cysteine residues in 47 proteins that have a high potential to be regulated in a reversible way by ROS. Strikingly, proteins of the cytosolic translational machinery were overrepresented. Furthermore, we found H_2_O_2_-sensitive motifs in the cytosolic translation apparatus, including several bona fide components of the ribosome and translation factors. The involved redox-active cysteine residues are evolutionary conserved and coordinate a central Zn^2+^ ion in a tetrahedral configuration. Interestingly, structurally related zinc-binding sites in Hsp33, also featuring a tetrahedral geometry, were shown to serve as redox sensors of high levels of ROS in vivo^49,50^.

The synthesis of proteins is vital for cell growth and proliferation. Global cytosolic translation is known to be regulated under various stress conditions such as protein misfolding stress in the endoplasmic reticulum, UV irradiation, amino acid starvation, hypoxia, viral infections and oxidative stress^32–39^. In addition, H_2_O_2_ treatment of cells inhibits TOR signalling, which can contribute to translation reduction. However, our results show that oxidative stress itself is sufficient to block cytosolic translation and this can be reversed once physiological conditions are restored. This suggests a mechanism that cells utilise to rapidly respond to changes in physiological and pathological conditions in order to adjust protein synthesis rates. Moreover, this translational response exists not only in yeast, but also in higher eukaryotic cells, which is in line with the evolutionary conservation of translational redox switches identified in this study. In further support of our proposal, the treatment of yeast cells with H_2_O_2_ was previously shown to result in global and reversible translation reduction^36,40,51^. However, the link between endogenously produced ROS and attenuation of protein synthesis has remained unclear.

The majority of ROS within the cell is produced by mitochondria and several defects in mitochondrial function result in ROS production. On the one hand, a study in *Caenorhabditis elegans* raised the possibility that increased mitochondrial ROS levels may influence cytosolic translation^38^. On the other hand, we showed earlier that cells defective in the mitochondrial protein biogenesis activate a response that includes robust attenuation of cytosolic protein synthesis^27^. We now show that the inhibitory effect on translation was caused by increased ROS that are overproduced due to inefficient mitochondrial protein biogenesis. The redoxome analysis of yeast with elevated mitochondrial ROS production and decreased cytosolic translation revealed changes in the redox status of various proteins important for the synthesis of proteins. Thus, mechanisms underlying translation modulation mediated by oxidative changes in proteins appear to be more complex under endogenously elevated ROS levels originating from active mitochondria. Cells use ROS species to signal endogenous stress derived from mitochondrial dysfunction to regulate cytosolic translation in a reversible manner. Our data suggest that the translation apparatus is equipped with conserved, redox-sensitive switches to directly respond to increased levels of ROS, a hallmark of defective mitochondria, by decreasing the load of newly synthesised proteins. Ribosomal proteins can act as sensors for oxidative stress mediating a decrease in protein synthesis by pausing the translation at the post-initiation stage. This is in agreement with our previous findings demonstrating that despite global translation reduction in yeast with defective mitochondria polysomes are maintained^27^. Our data show that depletion of ribosomal proteins partially prevented translation attenuation. It is also plausible that the cells depleted of certain ribosomal proteins undergo an adaptation, which results in higher oxidative stress tolerance. Therefore, more work will be needed to decipher exact molecular changes in the translation machinery that ultimately result in a halt in protein synthesis.

Our work identifies a novel mechanism of translational inhibition that involves ROS signalling upon mitochondrial stress. Knowledge about reversible redox changes mediating the adjustment of global protein synthesis provides important implications in understanding consequences of mitochondrial pathologies.

## Methods

### Yeast strains

Yeast, *S. cerevisiae*, strains are derivatives of BY4741 (*MATa, his3Δ1; leu2Δ0; met15Δ0; ura3Δ0*) or YPH499 (*MATa, ade2-101, his3-Δ200, leu2-Δ1, ura3-52, trp1-Δ63, lys2-801*). The *mia40-4int* (Fomp2-7int; 305), *mia40-4* (YPH-fomp2-7; 176), *mia40-3* (YPH-BG-fomp2-8; 178), *mia40*-F311E (660) strains and the corresponding wild-type strain (398) were described previously^24,52,53^. The deletion strains of *RPS29A*, *RPS29B*,* RPL40A*,* RPL40B*, *RPL26A*, *RPL26B*, *RPL34A*, *RPL34B*, *RPL37A*, *RPL37B*,* TOR1*, *SCH9* and *TIF3* in the BY4741 genetic background were purchased from Open Biosystems. To express *RPL40A* (YIL148W) wild-type, *RPL40A*-C115S and *RPL40A*-C115D in frame with haemagglutinin tag under endogenous promoter and terminator, wild-type (BY4741) yeast cells were transformed with centromeric LEU2-containing pUT11 (603), pUT12 (604) and pUT13 (605) plasmids. *RPL40A* was amplified by PCR from yeast genomic DNA including 552 bp of the 5’-untranslated region (5’UTR) and 493 bp of the 3’UTR. Cysteine mutants of *RPL40A*, C115S and C115D were generated by site-directed mutagenesis.

### Yeast growth conditions and mitochondrial procedures

For OxICAT analysis, YPH499 strain was grown in three biological replicates at 28 °C on minimal synthetic medium (0.67% (w/v) yeast nitrogen base, 0.079% (w/v) CSM amino acid mix) containing 2% (v/v) galactose to OD600 about 0.6 and treated or not with 1 mM H_2_O_2_ (H1009, Sigma) for 30 min. Temperature-sensitive Mia mutants were grown on minimal synthetic medium (0.67% (w/v) yeast nitrogen base, 0.079% (w/v) CSM amino acid mix) containing 3% (v/v) glycerol supplemented with 0.05–0.2% (v/v) glucose. Mia mutants were grown at 19 °C or shifted to restrictive temperature of 37 °C. For OxICAT analysis, *mia40-4int* and corresponding wild-type (YPH499) yeast cells were grown in four biological replicates and shifted for 3 h to restrictive temperature (37 °C). At an OD600 of 0.8–1, cells were collected. For translation experiments, strains were grown on minimal synthetic medium (0.67% (w/v) yeast nitrogen base, 0.079% (w/v) CSM amino acid mix) containing 2% (v/v) glucose at 30 °C or YPG medium (1% (w/v) yeast extract, 2% (w/v) peptone, 3% (v/v) glycerol) at 28 °C. Cells were treated with various concentrations of H_2_O_2_ (H1009, Sigma), NAC (A9165, Sigma), ebselen (ALX-270-097, Enzo Life Sciences) and/or MG132 (BML-PI102, Enzo Life Sciences) when indicated. To treat cells with MG132, yeast were grown in modified YNB medium without ammonium sulphate but with 0.003% SDS. The samples were supplemented with either 25 µM MG132 or a corresponding volume of dimethyl sulphoxide (DMSO) as a solvent. For isolation of mitochondria, yeast strains were grown on YPG medium (1% (w/v) yeast extract, 2% (w/v) peptone, 3% (v/v) glycerol) or YPGal medium (1% (w/v) yeast extract, 2% (w/v) peptone, 2% (v/v) galactose). Mitochondria were isolated by differential centrifugation according to the previously described method^54^. Isolated mitochondria were resuspended in SM buffer (250 mM sucrose, 10 mM 3-(*N*-morpholino)propanesulfonic acid-KOH, pH 7.2). For Blue Native gel electrophoresis, mitochondria were resuspended in digitonin-containing buffer (1% [w/v] digitonin, 20 mM Tris-HCl, pH 7.4, 50 mM NaCl, 10% (w/v) glycerol, 0.1 mM EDTA, 1 mM phenylmethylsulphonyl fluoride (PMSF)) and the soluble fraction was separated on a 4–13% gradient gel at 4 °C. For OxICAT analysis, isolated mitochondria were resuspended in 10% TCA.

### Cell culture conditions

HEK 293 cells were obtained from ATCC. Cells were cultured in high glucose (4.5 g l^−1^) 90% Dulbecco’s modified Eagle medium (DMEM, Sigma D5671), supplemented with heat-inactivated (55 °C for 30 min) 10% fetal bovine serum), 2 mM l-glutamine, penicillin (100 U ml^−1^) and streptomycin (0.1 mg ml^−1^) at 37 °C in a humidified atmosphere of 5% CO_2_. The culture medium was changed every second day. 24 h before performing experiments, cells were cultured in 90% DMEM (Sigma D5030) medium with 10 mM galactose instead of glucose and the medium was supplemented as described above. Cells were treated with various concentrations of H_2_O_2_ (H1009, Sigma), NAC (A9165, Sigma), GSK2606414 (Tocris, 5107) and/ or INK128 (APExBIO, MLN0128) when indicated.

### Protein extraction for global redoxome and proteome analysis

Yeast cell pellets from 25 to 40 OD600 cultures were resuspended in 10% TCA, 150 mM NaCl and immediately frozen in liquid nitrogen. Samples were stored at −80 °C. They were homogenised by four cycles of 45 s bead beating (MiniLys, Peqlab) with glass beads of approximately 0.5 mm in diameter at maximum speed and dipping in liquid nitrogen between cycles. Supernatants were transferred to fresh reaction tubes and glass beads were washed twice with ice-cold 10% TCA solution adding the supernatants of the washing steps to each sample. Samples were mixed thoroughly by vortexing and 100 µl aliquots were removed for determination of protein concentration. The remaining sample was kept in 10% TCA on ice or at −20 °C.

### Determination of protein concentration for OxICAT samples

Aliquots in 10% TCA were pelleted by centrifugation (21,000 × *g*, 15 min, 4 °C). Pellets were washed once with 500 µl of 5% ice-cold TCA and then resuspended in 100 µl denaturing alkylation buffer (DAB, 6 M urea, 200 mM tris(hydroxymethyl)aminomethane-HCl (Tris-HCl), pH 8.5, 10 mM EDTA, 0.5% SDS). Protein concentration was estimated by Bradford assay with bovine serum albumin as standard.

### Differential thiol labelling and peptide enrichment

The thiol trapping approach was adapted from Leichert et al.^5^ and performed as follows: for OxICAT experiments, whole cell extracts in 10% TCA corresponding to 200 µg total protein, and isolated mitochondria in 10% TCA corresponding to 100 µg total protein were used. 200 µl of 100% acetonitrile (ACN) and 800 µl DAB were added to the content of a 10 unit vial of heavy ICAT reagent (ABSciex). Alternatively, 20 µl of 100% ACN and 80 µl DAB were added to the content of a 1 unit vial of heavy ICAT reagent. Samples in 10% TCA were pelleted by centrifugation (21,000 × *g*, 15 min, 4 °C). Pellets were washed once with 500 µl of 5% ice-cold TCA and immediately resuspended in 200 µl (whole cell extracts) or 100 µl (isolated mitochondria) of the heavy ICAT/ACN/DAB mixture. Reduced cysteine residues were labelled for 2 h at 37 °C under vigorous shaking. Cell debris was pelleted by centrifugation (21,000 × *g*, 1 min, 4 °C) and supernatants were diluted threefold with H_2_O to lower the urea concentration. For protein precipitation, samples were incubated with 5 volumes of ice-cold acetone for at least 2 h at −20 °C and then pelleted by centrifugation for 30 min at 4,500 × *g* and 4 °C. Pellets were washed once with 100% ice-cold acetone and re-solubilised in 80 µl DAB. Mitochondria samples were supplemented with the contents of a 1 unit vial of light ICAT reagent dissolved in 20 µl of 100% ACN. Subsequently, tris(2-carboxyethyl)phosphine (TCEP) was added to a final concentration of 1 mM. For whole-cell extract samples, TCEP was added to a final concentration of 2.5 mM and these samples were pre-incubated for 5 min at 37 °C, whereas a mixture of light ICAT, ACN and DAB was prepared by adding 200 µl of 100% ACN and 400 µl DAB to the contents of a 10 unit vial of light ICAT reagent. One hundred and twenty microlitres of this mixture were added to each whole cell extract sample (final TCEP concentration 1 mM). All samples were incubated for 2 h at 37 °C under vigorous shaking. Subsequently, samples were diluted threefold with H_2_O followed by precipitation of proteins by incubation of samples in five volumes of ice-cold acetone for at least 2 h at −20 °C. Precipitated proteins were pelleted at 4,500 × *g* at 4 °C for 30 min. For tryptic digestion of OxICAT-labelled proteins from whole cell extracts, 200 µl of 20 mM ammonium bicarbonate (NH_4_HCO_3_) were added to each sample followed by incubation for 20 min at 4 °C in an ultrasonic bath. Trypsin (sequencing-grade modified, Promega; or MS approved, SERVA) was reconstituted with Trypsin Resuspension Buffer (50 mM acetic acid, Promega) to a final concentration of 0.5 µg µl^−1^. To each sample, 12 µl trypsin solution were added (trypsin : protein ratio of 1 : 30) and samples were incubated at 37 °C for 16 h. Samples were pelleted by centrifugation (21,000 × *g*, 1 min, room temperature (RT)). Supernatants were transferred to fresh reaction tubes, dried in vacuo and stored at −80 °C. Pellets (i.e., proteins insoluble in 20 mM NH_4_HCO_3_) were resuspended in 50 µl of 20 mM NH_4_HCO_3_, 60% (v/v) methanol. Afterwards, 2 µl of trypsin solution were added, samples were incubated at 42 °C for 3 h, dried in vacuo and stored at −80 °C. For tryptic digestion of OxICAT-labelled mitochondrial samples, one vial of trypsin with CaCl_2_, provided with the ICAT kit (ABSciex), was resuspended in 200 µl H_2_O. One hundred microlitres of the trypsin solution and 150 µl methanol were added to each mitochondrial sample. Mitochondrial samples were incubated at 37 °C for 16 h, dried in vacuo and stored at −80 °C.

Tryptic digests were subjected to strong cation exchange (SCX) chromatography before enrichment of OxICAT-labelled peptides. To this end, dried peptides of whole-cell extract samples from tryptic digests in 20 mM NH_4_HCO_3_ and in 20 mM NH_4_HCO_3,_ 60% methanol were each resuspended in 2.5 ml of SCX loading buffer (10 mM monopotassium phosphate KH_2_PO_4_), 25% (v/v) ACN, pH 3.0 adjusted with phosphoric acid) and then combined. Dried peptides of mitochondrial samples were resuspended in 4 ml SCX loading buffer. Cation exchange cartridges (provided with the ICAT kit) were equilibrated with 2 ml SCX loading buffer. Subsequently, samples were loaded, washed with 1 ml SCX loading buffer and eluted with 500 µl SCX elution buffer (10 mM KH_2_PO_4_, 25% (v/v) ACN, 350 mM KCl, pH 3.0). Cartridges were cleaned using 2 ml SCX washing buffer (10 mM KH_2_PO_4_, 25% (v/v) ACN, 1 M KCl, pH 3.0). For the enrichment of OxICAT-labelled peptides, samples were then subjected to avidin affinity chromatography. To this end, 500 µl affinity loading buffer (20 mM NaH_2_PO_4_, 300 mM NaCl, pH 7.2) were added to each eluate. Avidin affinity cartridges (provided with the ICAT kit) were cleaned with 2 ml affinity elution buffer (30% (v/v) (ACN, 0.4% (v/v) trifluoroacetic acid (TFA)) and then equilibrated with 2 ml affinity loading buffer. Next, samples were loaded and washed with 500 µl SCX loading buffer, 1 ml affinity washing buffer 1 (10 mM NaH_2_PO_4_, 150 mM NaCl, pH 7.2), 1 ml affinity washing buffer 2 (50 mM NH_4_HCO_3_, 20% (v/v) methanol) and 1 ml H_2_O. Peptides were then eluted with 800 µl affinity elution buffer with discarding of the first 50 µl of the eluate and the cartridge was re-used for the next sample. Eluates were dried in vacuo. To remove the biotin tag of the ICAT labels, samples were re-dissolved in 90 µl of a mixture of 95% (v/v) cleaving reagent A (100% TFA) and 5% (v/v) cleaving reagent B (provided with the ICAT kit, ABSciex) and incubated for 2 h at 37 °C with vigorous shaking. Samples were dried in vacuo and stored at −80 °C. For LC-MS/MS analysis, peptide samples from whole-cell extracts and isolated mitochondria were resuspended in 30 µl and 15 µl of 0.1% TFA, respectively.

### Reduction and alkylation of proteins and tryptic digestion

Whole-cell extracts of control and H_2_O_2_-treated samples, each corresponding to 100 µg total protein in 10% TCA, were pelleted by centrifugation (21,000 × *g*, 15 min, 4 °C). Pellets were washed once with 500 µl of 5% ice-cold TCA and resuspended in 100 µl of 6 M urea, 5 mM EDTA, 200 mM Tris-HCl, pH 8. Cysteines were reduced by addition of TCEP to a final concentration of 10 mM and samples were incubated for 30 min at 37 °C under vigorous shaking. Cysteine residues were subsequently alkylated by addition of chloroacetamide to a final concentration of 50 mM and incubation for 30 min at RT with vigorous shaking. To quench the reaction, dithiothreitiol (DTT) was added to a final concentration of 20 mM. To remove cell debris, samples were pelleted by centrifugation (21,000 × *g*, 1 min, RT) Supernatants were diluted fourfold with H_2_O. For tryptic digestion of proteins, one vial sequencing grade modified trypsin (Promega) was reconstituted with 40 µl Trypsin Resuspension Buffer (50 mM acetic acid, Promega). To each sample, 4 µl trypsin solution were added (trypsin : protein ratio of 1 : 50) and samples were incubated at 42 °C for 4 h and then desalted using C18-SD 7 mm per 3 ml extraction disc cartridges (Empore 3 M). To this end, cartridges were conditioned by subsequent addition of 1 ml of 100% methanol, 500 µl solution B (70% (v/v) ACN, 0.1% (v/v) TFA) and 500 µl solution A (0.1% TFA), each applied by centrifugation for 1 min at 350 × *g*. Samples were acidified by addition of 4 µl of 100% TFA, then loaded onto a conditioned cartridge by centrifugation for 4 min at 200 × *g*, washed once with 500 µl solution A by centrifugation for 3 min at 200 × *g*, and finally eluted in 500 µl ACN by centrifugation for 2 min at 200 × *g*. Eluates were dried in vacuo.

### Dimethyl labelling of peptides and fractionation

Stable isotope dimethyl labelling of peptides was performed in solution essentially as described previously^20^. In brief, each sample was reconstituted in 337.5 µl of 100 mM triethylammonium bicarbonate buffer and 15 µl of 4% (v/v) formaldehyde. Subsequently, 15 µl of 0.6 M cyanoborohydride were added to each sample with the light (CH_2_O and NaBH_3_CN, Sigma-Aldrich) and heavy (deuterated formaldehyde (CD_2_O) and deuterated cyanoborohydride (NaBD_3_CN), Sigma-Aldrich) isotopomeric versions being used for peptides from untreated (control) and H_2_O_2_-treated yeast cells, respectively. After 1 h incubation at RT with vigorous shaking, samples were placed on ice and the labelling reaction was quenched by addition of 60 µl of 1% (v/v) ammonia solution and acidified by 30 µl of formic acid (FA). Light- and heavy-labelled samples were mixed and desalted using C18-SD 7 mm per 3 ml extraction disc cartridges as described above, except that samples were not acidified by TFA. Following lyophilisation, each sample was reconstituted in 100 µl buffer A (10 mM ammonium hydroxide, pH 10), sonicated for 5 min and centrifuged (21,000 × *g*, 5 min, RT). The supernatant was filtrated using a 0.2 µm PTFE membrane syringe filter (Phenomenex) before peptides were separated by high pH reversed-phase chromatography using a Dionex Ultimate 3000 equipped with a NX 3u Gemini C18 column (150 mm × 2 mm, particle size 3 µM, pore size 110 Å, Phenomenex) at 40 °C with a flow rate of 200 µl min^−1^. The solvent consisted of 10 mM ammonium hydroxide, pH 10 as mobile phase A and 10 mM ammonium hydroxide, pH 10 in 90% (v/v) ACN as mobile phase B. Peptides were loaded at 1% B for 5 min and then separated using a linear gradient from 5–61% B in 55 min and 61–62% B in 2 min. Afterwards, the concentration of B was increased to 78% in 2 min and kept constant for 2 min. Ninety-six fractions were collected in a deep well plate from min 1.5 to min 70.2 at 43 s intervals and concatenated on-line into 32 samples by combining every thirty-second fraction. Samples were dried in vacuo and stored at −80 °C. Before LC-MS/MS analysis, the sample-containing wells of the plate were rinsed once with 120 µl of 86% ACN, 01% FA, the plate was placed on a horizontal shaker for 5 min and then dried in vacuo. Samples were resuspended in either 90 µl (32 samples of biological replicates 1 and 2) or 60 µl (32 samples of biological replicate 3) of 0.1% TFA and aliquots of 20 µl were analysed by LC-MS/MS.

### LC-MS analysis

Nano-high-performance liquid chromatography–electrospray ionisation (HPLC-ESI)–MS/MS analyses were performed on an Orbitrap Elite mass spectrometer (Thermo Scientific) for OxICAT samples and on a Q Exactive Plus mass spectrometer (Thermo Scientific) for dimethyl-labelled samples. Each instrument was connected to an UltiMate 3000 RSLCnano HPLC system (Thermo Scientific). Samples were washed and preconcentrated using PepMap C18 precolumns (length, 5 mm; inner diameter, 300 µm; Thermo Scientific) with a flow rate of 30 µl min^–1^ for 5 min (dimethyl-labelled samples and OxICAT samples from isolated mitochondria) or 25 min (OxICAT samples from whole cells). Peptide separation was performed using a C18 reversed-phase nano LC column (Acclaim PepMap RSLC column; length, 50 cm; inner diameter, 75 µm; particle size, 2 µm; pore size, 100 Å; Thermo Scientific) at 40 °C and a flow rate of 250 nl min^−1^. OxICAT samples from whole cells (three biological replicates for H_2_O_2_-treated samples and the respective untreated controls or 4 biological replicates for *mia40-4int* samples and the respective wild-type controls) were analysed in two technical replicates by LC-MS/MS. To this end, peptides were separated using a binary solvent system consisting of 0.1% (v/v) FA, 4% (v/v) DMSO (solvent A) and 48% methanol, 30% ACN, 0.1% (v/v) FA, 4% (v/v) DMSO (solvent B). They were eluted with a gradient of 5–25% B in 85 min, 25–45% B in 80 min, 45–60% B in 50 min, 60–80% B in 20 min and 80–99% B in 10 min. Subsequently, the analytical column was washed with 99% B for 5 min before re-equilibration with 95% A. OxICAT samples from isolated mitochondria were separated using a binary solvent system consisting of 0.1% (v/v) FA (solvent A) and 50% methanol, 30% ACN, 0.1% (v/v) FA (solvent B). They were eluted with a gradient of 10–15% B in 20 min, 15–65% B in 125 min and 65–99% B in 10 min. Then the analytical column was washed with 99% B for 5 min before re-equilibration with 90% A. Dimethyl-labelled peptide samples from whole cells (*n* = 3 biological replicates) were separated using a binary solvent system consisting of 0.1% (v/v) FA, 2% (v/v) DMSO (solvent A) and 86% ACN, 0.1% (v/v) FA, 2% (v/v) DMSO (solvent B). They were eluted with a gradient of 3–39% B in 30 min and 39–95% B in 5 min. Then the analytical column was washed with 95% B for 5 min before re-equilibration with 97% A. On the Orbitrap Elite, peptides eluting from the column were directly transferred to a stainless steel emitter (Thermo Scientific) via a DirectJunction adaptor (Thermo Scientific) for ESI (spray voltage 1.8 kV, temperature of heated capillary 200 °C) using a nanospray flex ion source (Thermo Scientific). Mass spectra were acquired in a mass-to-charge (*m/z*) range of 370–1,700 with a resolution (*R*) of 120,000 at *m/z* 400. Automatic gain control (AGC) was set to 1 × 10^6^ with a maximum (max.) injection time of 200 ms. The 15 most intense peptide peaks were selected for low-energy collision-induced dissociation experiments in the linear ion trap with the following parameters: normalised collision energy (CE), 35%; activation *q*, 0.25; activation time, 10 ms; AGC, 5,000; max. injection time, 150 ms; isolation width, *m/z* 2.0.

On the Q Exactive Plus system, peptides eluting from the LC column were directly transferred to a fused silica emitter (PicoTip; length, 5 cm; inner diameter, 10 µm; NewObjective) for ESI (spray voltage, 1.5 kV; heated capillary, 200 °C) using a nano-electrospray source (Proxeon). MS spectra were acquired in an *m/z* range of 375–1,700 with *R* of 70,000 at *m/z* 200. AGC was set to 3 × 10^6^ with a max. injection time of 60 ms. The 12 most intense peptide peaks were selected for higher-energy collisional dissociation (normalised CE, 28%) experiments with the following parameters: *R*, 35,000 at *m/z* 200; AGC, 1 × 10^5^; max. injection time, 120 ms; isolation width, *m/z* 3.0; underfill ratio, 0.7%.

Both instruments were operated in the positive ion mode for data-dependent acquisition of MS/MS spectra. The dynamic exclusion time of previously selected precursor ions was set to 45 s and only +2 or higher charged ions were selected for MS/MS scans.

### MS data analysis

For peptide identification in OxICAT experiments, Andromeda^55^ integrated in MaxQuant 1.4.1.2^56^ was used to search peak list against the *S. cerevisiae* proteome (UniProtKB canonical set for strain AC204508/S288c, Proteome ID UP000002311, release 23.01.2014, 6,643 entries). ICAT light tag (C_10_H_17_N_3_O_3_, 227.13 Da) and ICAT heavy tag (^13^C_9_CH_17_N_3_O_3_, 236.16 Da) were set as light and heavy label, respectively, with cysteine residue specificity. Up to five labelled amino acids per peptide were allowed. Trypsin/P was set as the enzyme in specific digestion mode allowing one missed cleavage site. The precursor mass tolerance was set to 20 p.p.m. for the ‘first search’ option of Andromeda and to 4.5 p.p.m. for the main search. No variable or fixed modifications were considered. Minimum peptide length was set to six amino acids and the minimum score for peptide identification to 40. A peptide spectrum match false discovery rate (FDR) of 1% was applied using the decoy mode ‘Revert’. The options ‘Re-quantify’, ‘Match between runs’, ‘Second peptides’ and ‘Dependent peptides’ were left unchecked. Filtering for labelled amino acids was enabled. For MS raw data from whole-cell extract samples, the two technical replicates of each biological replicate (‘Experiment’) were defined as fraction 1 and 2, respectively, in the experimental design template. MS raw data from H_2_O_2_-treated samples and respective untreated control samples were jointly processed. Likewise, MS raw data of *mia40-4int* samples and the respective wild-type control samples were jointly processed. MS raw data of the two samples form isolated mitochondria were jointly processed with raw data of two additional mitochondrial samples that were treated with 1 mM diamide, but otherwise prepared identically. Identified peptides with a posterior error probability (PEP) ≥ 0.0105, cysteine-containing peptides with zero intensity and decoy (‘REV’) entries were removed from the respective ‘peptides.txt’ result tables. Peptide quantification of OxICAT samples was performed using Skyline 2.5.0 (https://skyline.gs.washington.edu)^16^. For data import in Skyline, ‘msms.txt’ of the MaxQuant search was trimmed to cysteine-containing peptides with a PEP value of < 0.0105 and an intensity > 0. Decoy entries were removed. For whole-cell extracts of H_2_O_2_-treated and untreated control samples, ‘msms.txt’ was further narrowed down to peptides identified with ≥ four evidences of the same charge state (each identified with a PEP value of < 0.0105 and an intensity > 0) throughout the dataset. For the *mia40-4int* dataset, ‘msms.txt’ was restricted to peptides identified in at least three out of four biological replicates in both *mia40-4int* and wild-type samples, in each biological replicate identified with a PEP value of < 0.0105 and an intensity > 10^6^. For isolated mitochondria samples, only peptides identified in at least three out of four samples were included in ‘msms.txt’ for import in Skyline. If a cysteine residue or a combination of cysteine residues was represented by different peptides in the dataset (due to identification of the peptide with or without missed cleavage sites), usually only one peptide sequence was imported in Skyline. To that end, each cysteine-containing peptide was assigned a CysID composed of the Uniprot identifier of the leading razor protein and the position(s) of the cysteine residue(s) in the protein sequence of the leading razor protein as stored in the FASTA file used for the MaxQuant search. Peptides with identical CysIDs were included into a preliminary Skyline analysis and total peak areas were extracted and summed up for each peptide to determine the peptide with the highest overall intensity. For datasets of H_2_O_2_-treated and mitochondrial samples, only the peptide with the highest intensity was selected for import in Skyline. For the samples of the *mia40-4int* dataset, generally the peptide with the highest intensity was chosen for import in Skyline but peptides representing identical CysIDs of lower intensity were not excluded from Skyline import if they were already present in the H_2_O_2_ dataset. The information regarding which peptides were chosen for quantification by Skyline can be found in Supplementary Data 2 (H_2_O_2_ dataset), Supplementary Data 3 (isolated mitochondria) and Supplementary Data 7 (*mia40-4int* dataset). Peptide settings used for Skyline import were matched to MaxQuant search parameters (enzyme, trypsin [KR│P]; max. missed cleavages, 1; min. peptide length, 6 amino acids (aa); modification, ICAT-C; isotope modification, ICAT-C13). Maximum peptide length was set to 44 aa in agreement with identification results. For import of data from mitochondrial samples, the precursor isotopic import filter was set to a count of three (M, M + 1 and M + 2) at a resolution of 100,000 at *m/z* 400 and only scans within a 5 min-window of the predicted retention time for a peptide were extracted. Whole-cell extract data were analysed employing two separate Skyline documents for each of the two datasets: for short peptides (length ≤ 20 aa), the first two isotope peaks (M and M + 1) and, for long peptides (> 20 aa), the first three isotope peaks (M, M + 1 and M + 2) were chosen, both at a resolution of 100,000 at *m/z* 400 and only scans within a 2 min window of MS/MS IDs were imported. Graphical displays of chromatographic traces (extracted ion chromatograms, XICs) were inspected manually. When Skyline default peak picking failed, the borders for the integration of peak areas were adjusted manually. If a peptide ion was identified with more than one charge state, only the charge state of the peptide with the highest intensity was usually retained for quantification. Isotope peaks deviating in retention time were removed from both clusters before quantification. Isotope peaks having a mass error > 5 p.p.m. in one of the two isotope clusters were excluded from quantification in the H_2_O_2_ dataset and the mitochondrial samples. Isotope peaks deviating 5 p.p.m. from the average mass error in one of the two isotope clusters were excluded from quantification in the *mia40-4int* dataset. When the retention time profiles of ‘light’ and ‘heavy’ peaks were inconsistent altogether, the respective XICs were also excluded from quantification (denoted by ‘nq’ in Supplementary Data 2, 3, and 5). For the remaining peptides comprising identical CysIDs in the *mia40-4int* dataset, the peptide sequence already quantified in the H_2_O_2_ dataset was chosen for quantification. Total MS1 peak areas were exported for each isotope label type and technical replicate for each peptide sequence. Using Excel (v. 2010), total MS1 peak areas of technical replicates 1 and 2 were summed up and then the proportion of reversibly oxidised (^12^C-ICAT-labelled) cysteine residues (% oxidation) was calculated for each biological replicate and peptide sequence by the formula $\frac{{{\mathrm{intensity}}\,{\mathrm{light}}}}{{{\mathrm{intensity}}\,{\mathrm{light}} + {\mathrm{intensity}}\,{\mathrm{heavy}}}} \times 100$, where intensity light is the sum of total MS1 peak areas of the isotope label type ‘light’ of technical replicate 1 and 2 and intensity heavy is the sum of total MS1 peak areas of isotope label type ‘heavy’ of technical replicate 1 and 2. Mean % oxidation values were calculated if at least two out of three biological replicates of the H_2_O_2_ dataset or at least three out of four biological replicates of the *mia40-4int* dataset were quantified. SD was calculated by $\sqrt {{\sum} {\frac{{(x - {\bar{x}})^2}}{{\left( {n - 1} \right)}}} }$, where *x̅* is the sample mean and *n* is the sample size. Analysis of variance was calculated using Perseus (v.1.4.0.8). Artificial within groups’ variance was set to zero and the *P-*value was used for truncation with a threshold of 0.05.

MS raw data of dimethyl-labelled samples were jointly processed by MaxQuant 1.5.3.12^56^. For protein identification, spectra were correlated with the *S. cerevisiae* protein database (UniProtKB canonical set including isoforms for strain AC204508/S288c, Proteome ID UP000002311, release 01.10.2015, 6,740 entries) using Andromeda^55^. N-terminal and lysine dimethylation were set as light (+28.03 Da) and heavy (+34.06 Da) labels, respectively. Trypsin/P was set as the enzyme in specific digestion mode allowing up to two missed cleavage sites. Oxidation of methionine was included as variable modification and cysteine carbamidomethylation was set as fixed modification. The precursor mass tolerance was set to 20 p.p.m. for the first and to 4.5 p.p.m. for the main search. Minimum peptide length was kept at 7. A FDR of 1% was applied on both peptide and protein lists using the decoy mode ‘Revert’. For protein quantification, the option ‘Re-quantify’ was checked and the option ‘Match between runs’ was enabled with a retention time window of 0.7 min. The minimum ratio count for protein quantification was set to two and only unique peptides were considered for protein quantification. Normalised light-over-heavy ratios of the MaxQuant output file ‘proteingroups’.txt were log_10_-transformed, mean log_10_ ratios across all three replicates were calculated, and the *P-*value for each protein was determined using a two-sided *t*-test implemented in Perseus (v.1.4.0.8)^57^. The data were normally distributed, thus meeting the assumption of the statistical test. Only proteins identified with ≥ 2 peptides (at least one of them unique) in the entire dataset that were quantified by MaxQuant in ≥ 2 biological replicates were considered for statistical analysis.

### Analysis of disulfide bond and zinc-binding annotations

Known and predicted disulfide bond annotations were retrieved from the Uniprot database (on 19.05.2015) and the positions of disulfide bond-forming cysteine residues within the protein sequence were matched to the respective positions of cysteine residues within the tryptic peptides. In the case of peptides containing more than one cysteine residue, a disulfide bond annotation was assigned if at least one cysteine residue within the peptide was annotated as disulfide bond-forming in the database. The annotations ‘transient disulfide bond’, ‘disulfide bond in linked form’ and ‘disulfide bond in nuclear retained form’ were summarised as ‘other disulfide form’.

Zinc-binding and zinc finger annotations were extracted for each protein from the Uniprot database (on 19.05.2015). The positions of zinc-binding cysteine residues within the protein sequence were matched to the respective positions of cysteine residues within the tryptic peptide. In the case of peptides containing more than one cysteine residue, a zinc-binding annotation was assigned, if at least one cysteine residue within the peptide was annotated as zinc-binding in the database. For zinc finger annotations, the positions of the first and the last amino acid of the zinc finger were noted for each protein. A zinc finger annotation was assigned to a peptide, if it contained at least one cysteine residue, which position fell into the range of the zinc finger.

### Structural analysis of H_2_O_2_-sensitive sequences

The sequence motif of H_2_O_2_-sensitive sequences (≥ + 7% oxidation, *P-*value < 0.05, *n* = 3) was visualised by Weblogo 2.8.2 (http://weblogo.berkeley.edu/)^58^. The images of the crystal structures of 4u4r^59^, 3j6b^60^ and 3jap^61^ were created using PyMOL (The PyMOL Molecular Graphics System, Version 1.3 Schrödinger, LLC; http://www.pymol.org/).

### Sequence alignments

The Basic Local Alignment Search Tool (BLAST) implemented in UniProt (http://www.uniprot.org/blast/) was employed to identify homologues of the yeast proteins Rps26, Rps29, Rpl37, Rpl40, Mrpl32 and Sui3, respectively, by consecutively entering the respective UniProt identifiers (P39939, P41058, P49166, P0CH09, P25348 and P09064) in the form field. The target database was set to Vertebrates to identify *Homo sapiens*, *Mus musculus* and *Danio rerio* homologues, to Arthropoda to identify *Drosophila melanogaster* homologues, and to Nematoda to identify *C. elegans* homologues for each yeast protein. All BLAST searches were conducted with default programme parameters and in each case the top hit of reviewed entries was chosen for multiple sequence alignments. Multiple sequence alignments were constructed using Clustal O (1.2.1)^62^ implemented in UniProt (http://www.uniprot.org/align/).

### Protein association network

STRING^63^ (version 10.0) was used to generate a functional protein association network using the search form for multiple proteins. The minimum required interaction score was set to highest confidence (0.9). The meaning of network edges was set to the term “confidence” (line thickness indicates strength of data support). Structure previews inside network edges were disabled. The resulting network was exported as vector graphic, nodes were recoloured and placed symmetrically.

### GO analysis

To analyse the cellular localisation of proteins, all quantified cysteine-containing proteins were sorted according to the GO Slim terms provided by the *Saccharomyces* genome database. Proteins with several peptides in different oxidation levels were grouped in the oxidation range of the most oxidised peptide.

To analyse the overrepresentation of GO terms, the free web tool ‘Ontologizer’ was used^64^. All quantified cysteine-containing proteins within the respective range of oxidation were compared to the entire dataset of quantified cysteine residue-containing proteins. The built-in Benjamini–Hochberg procedure was used to correct raw *P-*values calculated for GO enrichment analysis for multiple testing. GO terms with a corrected *P-*value < 0.05 were considered enriched.

### Biochemical analysis of the redox state of proteins

Crude cell extracts were prepared by incubation of six OD600 units of yeast cells in 10% TCA. Cells were disrupted using glass beads. The supernatant was centrifuged at 6,000 × *g* for 15 min at 4 °C to pellet the TCA precipitate. The TCA precipitate was washed once with ice-cold acetone and resuspended in DA-buffer (6 M urea, 0.2 M Tris-HCl pH 7.4, 10 mM EDTA, 0.5% SDS). The sample was split into two equal aliquots. 10 mM iodoacetamide (IAA) was added to one aliquot to alkalise reduced cysteines. Both aliquots were incubated for 1 h at 30 °C. Both aliquots were split again, each into two equal aliquots. Cysteine residues were reduced by adding 50 mM TCEP to one aliquot treated before with IAA and one aliquot not treated with IAA. All aliquots were incubated for 15 min at 37 °C. The concentration of urea was lowered to at least 1.5 M by adding ddH_2_O and proteins were precipitated with five volumes of acetone overnight at −20 °C. Precipitates were centrifuged at 6,000 × *g* for 30 min at 4 °C, washed once with ice-cold acetone and resuspended in Laemmli buffer. To aliquots previously treated with TCEP either 10 mM 4-acetamido-4’-maleimidylstilbene-2,2’-disulfonic acid (A481, Molecular Probes) or 10 mM methyl-PEG_24_-maleimide (22713, Thermo Scientific) were added. All aliquots were incubated for 45 min at 30 °C. To the aliquot treated previously with IAA only, 10 mM IAA was added and to the previously untreated aliquot, 100 mM DTT were added. Proteins were denatured for 15 min at 65 °C and analysed by western blotting.

### Translation assays

Yeast strains were grown on selective minimal synthetic medium (0.67% (w/v) yeast nitrogen base, 0.75% (w/v) -Met amino acid mix) containing 2% (v/v) glucose (for treatment with H_2_O_2_) or 3% (v/v) glycerol (for *mia40* mutants). Proteins were radiolabelled by adding [^35^S] methionine and [^35^S] cysteine (EasyTag EXPRESS35S Protein Labelling Mix, Perkin Elmer) at a final concentration of 6 μCi ml^−1^. The *mia40* mutant cells were labelled for 1 h. Yeast cells were collected and washed once with ddH_2_O. HEK 293 cells were grown to 70–90% confluence. Cells were washed twice with phosphate buffered saline (PBS) and incubated at 37 °C for 60 min in Roswell Park Memorial Institute medium without methionine and serum (R7513, Sigma). Proteins were radiolabelled for 1 h at 37 °C. Cells were placed on ice, collected and washed three times with ice-cold PBS. Yeast protein extracts or extracts from mammalian cells were separated by SDS-PAGE and radiolabelled proteins were detected with Typhoon TRIO + Variable Mode Imager (GE Healthcare).

### ROS measurements and carbonylation assays

To analyse the levels of ROS production, cultures were grown to OD600 0.5–0.9. The yeast cells corresponding to one OD600 were collected and washed with PBS (137 mM NaCl, 12 mM phosphate, 2.7 mM KCl, pH 7.4). The yeast cells were incubated for 40 min at RT in PBS containing CM-H_2_DCFDA dye for measuring endogenous levels of H_2_O_2_ or dihydroethidium dye, to measure endogenous levels of superoxide anion. Fluorescent signals were corrected for auto-fluorescence without respective dye. Fluorescence was measured for H_2_O_2_ at an excitation wavelength of 488 nm and an emission wavelength of 533 nm and for superoxide anions at an excitation wavelength of 535 nm and an emission wavelength of 635 nm using a plate fluorometer (Infinite M1000, Tecan). The method for the analysis of protein carbonylation was adapted from Levine et al.^65^. Crude cell extracts were prepared by incubation of 20–30 OD600 units of yeast cells in lysis buffer (20 mM Tris-HCl pH 7.4, 200 mM NaCl, 0.5% Triton X-100, 1 mM PMSF). Cells were disrupted using glass beads. The supernatant was cleared by centrifugation at 3,000 × *g* for 1 min. To precipitate DNA in the protein lysate, 10% streptomycin in 50 mM 4-(2-hydroxyethyl)−1-piperazineethanesulfonic acid pH 7.0 was added to a final concentration of 1% and incubated for 15 min on ice followed by centrifugation at 6,000 × *g* for 10 min at 4 °C. The lysate was split into two equal aliquots. To one aliquot, 4 volumes of 10 mM 2,4-dinitrophenylhydrazine diluted in 2 N hydrochloric acid (HCl) were added. To the other aliquot, four volumes of 2 N HCl were added (control). The samples were incubated for 1 h at RT with occasional vortexing. Proteins were precipitated by adding 20% TCA to a final concentration of 10% followed by incubation on ice for 30 min and centrifugation at 20,000 × *g* for 15 min at 4 °C. The pellets were washed three times with an ice-cold 1:1 (v/v) mixture of ethanol and ethyl-acetate and resuspended in 8 M urea. Carbonylated proteins were detected by immunoblotting using anti-DNP antibody or quantified by spectrophotometry at 370 nm absorbance.

### Cell viability assays

One OD600 unit of yeast cells was collected and the pellet was washed once with PBS. Yeast cells were stained with 3 µg/ml propidium iodide in PBS for 15 min at RT protected from light. As a negative control, an aliquot of each sample was kept untreated. Samples were kept on ice and analysed within 1 h by flow-cytometry (FACSCalibur). Ten thousand cells per each sample were measured. HEK 293 cells were mixed with an equal volume of 0.4% Trypan Blue solution (T8154, Sigma) and incubated for 2 min at RT. Viable cells (unstained) were counted using a hemocytometer and a light microscope (Opta-Tech).

### Statistical analysis

Number of sample size, replicates, controls and statistical tests were chosen according to published data with comparable methodology and generally accepted standards in the field. Accordingly, no statistical method was used to predetermine sample size. For statistical analysis of ROS-, carbonylation measurements and survival assays a two-sided, paired *t*-test was performed. Data are presented in fold change compared with wild-type strain or in case of treatment with H_2_O_2_ were compared with mock-treated control sample. For quantification of translation experiments presented in Fig. 6d, e and Supplementary Fig. 5f, g, we used GelQuant.NET software provided by biochemlabsolutions.com. All experiments including western blot analyses have been replicated at least three times.

### Miscellaneous

Total protein extracts of yeast cells were prepared by alkaline lysis^66^ and of mammalian cells were prepared in RIPA buffer (50 mM Tris-HCl pH 7.5, 150 mM NaCl, 1% Triton X-100, 0.5% sodium deoxycholate, 0.1% SDS, 1 mM EDTA, 2 mM PMSF), respectively. Laemmli buffer containing 50 mM DTT was added and proteins were denatured at 65 °C for 15 min. Total protein extracts were separated by SDS-PAGE on 15% or 12% gels. Antibodies (dilution 1 : 500) against yeast proteins were raised in rabbits, used in previous studies, and controlled for specificity. Commercially available antibodies against yeast proteins (Rpt5 (PW8245, Enzo Life Science, dilution 1 : 10,000), Rpt1 (PW8255, Enzo Life Science, dilution 1 : 10,000)) or against mammalian proteins (TIMM23 (611222; BD Biosciences, dilution 1 : 500), SDHA (D-4) (sc-1669IA47; Santa Cruz, dilution 1 : 800), HSP70 (ADI-SPA-812; Enzo Life Sciences, dilution 1 : 500), RPL7 (A300-741A; Bethyl, dilution 1 : 2,000), RPL26 (A300-686A; Bethyl, dilution 1 : 2,000), 4E-BP1 (9644; Cell Signalling Technology, dilution 1 : 500), Phopho-4E-BP1 (9451; Cell Signalling Technology, dilution 1 : 500), eIF2α (9722; Cell Signalling Technology, dilution 1 : 500), Phospho-eIF2α (9721; Cell Signalling Technology, dilution 1 : 250), Phospho-eIF2α (Sui2) (ab32157; Abcam, 1 : 1,000)) were used. Protein bands were visualised using secondary anti-rabbit or anti-mouse antibodies conjugated with horseradish peroxidase and chemiluminescence. Chemiluminescence signals were detected by use of ImageQuant LAS4010 (GE Healthcare) or X-ray films. The images were processed digitally using Adobe Photoshop CS4. Full scans of western blottings and autoradiography are shown in Supplementary Information. Protein concentration was generally determined using Roti-Quant reagent (Carl Roth GmbH). For carbonylation assays, the protein amount was determined with the Micro BCA Protein Assay Kit (Thermo Scientific). Bovine serum albumin was used as a protein standard. In some figures, non-relevant gel parts were excised digitally.

### Data availability

OxICAT peptide identification and quantification data with the accompanying mass spectral evidence can be found in the respective Skyline documents that were uploaded to PanoramaWeb^67^ (https://panoramaweb.org/labkey/yeast_oxICAT.url). MS data of dimethyl-labelled samples have been deposited to the ProteomeXchange Consortium via the PRIDE partner repository^68^ with the dataset identifier PXD003584.

## Electronic supplementary material

Description of Additional Supplementary Files

Supplementary Information

Supplementary Data 1

Supplementary Data 2

Supplementary Data 3

Supplementary Data 4

Supplementary Data 5

Supplementary Data 6

Supplementary Data 7
