# Supplementary material for: Quantitative proteomics identifies redox switches for global translation modulation by mitochondrially produced reactive oxygen species
Source: Nat Commun. 2018 Jan 22;9:324. doi: 10.1038/s41467-017-02694-8 (PMC5778013; doi:10.1038/s41467-017-02694-8)
Supplement: Supplementary file 1 — Description of Additional Supplementary Files [file 41467_2017_2694_MOESM1_ESM.pdf]

Description of Additional Supplementary Files

File Name: Supplementary Data 1
Description: Peptides identified in OxICAT experiments of yeast cells. Reduced cysteine residues were labelled by heavy ICAT (^{13}C-ICAT). Reversibly oxidised cysteine residues were reduced by TCEP and labelled by light ICAT (^{12}C-ICAT). ICAT-labelled peptides were enriched by streptavidin affinity chromatography and analysed by LC-MS/MS. Mass spectrometric raw data of control and H_2O_2-treated samples were jointly processed by MaxQuant (v.1.4.1.2) for peptide identification. This sheet contains all peptide sequences identified with a posterior error probability (PEP) value < 0.01. Reverse entries were removed.

File Name: Supplementary Data 2
Description: *In vivo* oxidation status of cysteine-containing peptides of proteins from non-stressed yeast cells. MS1 ion chromatograms were extracted using Skyline (v.2.5.0). Integrated peak areas of the "heavy" (^{13}C-ICAT-labelled) and "light" (^{12}C-ICAT-labelled) version of the peptide ions were exported and the proportion of reversibly oxidised (^{12}C-ICAT-labelled) cysteine residues (% oxidation) was calculated. This sheet contains all cysteine-containing peptide sequences quantified in Skyline in at least two out of three biological replicates.

File Name: Supplementary Data 3
Description: Peptides identified in OxICAT experiments of isolated mitochondria. Mitochondria were isolated from either the yeast strain BY4741 or the yeast strain YPH499. Reduced cysteine residues were labelled by heavy ICAT (^{13}C-ICAT). Reversibly oxidised cysteine residues were reduced by TCEP and labelled by light ICAT (^{12}C-ICAT). ICAT-labelled peptides were enriched by streptavidin affinity chromatography and analysed by LC-MS/MS. Mass spectrometric raw data were processed by MaxQuant (v.1.4.1.2) for peptide identification. For selected peptides, MS1 ion chromatograms were extracted using Skyline (v.2.5.0). Integrated peak areas of the "heavy" (^{13}C-ICAT-labelled) and "light" (^{12}C-ICAT-labelled) version of the peptide ions were exported and the proportion of reversibly oxidised (^{12}C-ICAT-labelled) cysteine residues (% oxidation) was calculated. The table contains all cysteine-containing peptide sequences identified with posterior error probability (PEP) < 0.01 and intensity > 0. Reverse entries were removed. The mitochondrial reference set was adopted from previous work[9].

File Name: Supplementary Data 4
Description: Relative quantification of protein abundance changes upon H_2O_2 treatment. Proteins were extracted from control and H_2O_2-treated yeast cells and digested using trypsin. Tryptic peptides were labelled with "heavy" (H_2O_2) or "light" (control) isotopic versions of formaldehyde/cyanoborohydride. Light and heavy samples were mixed 1:1 based on total protein amount. Mixed samples were fractionated by high pH reversed-phase chromatography and analysed by LC-MS/MS.

Mass spectrometric raw data of all experiments were jointly processed by MaxQuant (v. 1.5.3.12) for protein identification and relative quantification. Normalized light-over-heavy ratios were log_{10}-transformed, mean log_{10} ratios across all three replicates were calculated, and the P value for each protein was determined using a two-sided t-test. All proteins listed in this table were identified with ≥ 2 peptides (at least one of them unique) in the entire dataset and were quantified by MaxQuant in ≥ 2 replicates.

File Name: Supplementary Data 5
Description: *In vivo* oxidation status of cysteine-containing peptides of yeast cells upon H_2O_2 treatment. MS1 ion chromatograms were extracted using Skyline (v.2.5.0). Integrated peak areas of the "heavy" (^{13}C-ICAT-labelled) and "light" (^{12}C-ICAT-labelled) version of the peptide ions were exported and the proportion of reversibly oxidised (^{12}C-ICAT-labelled) cysteine residues (% oxidation) was calculated for untreated (control, see Supplementary Table 2) and H_2O_2-treated samples. Analysis of variance (ANOVA) was performed between the group of samples exposed to H_2O_2 and the control group using Perseus (v.1.4.0.8). This sheet contains all cysteine-containing peptide sequences quantified in Skyline in ≥ 2 biological replicates of H_2O_2-treated samples and ≥ 2 biological replicates of control samples.

File Name: Supplementary Data 6
Description: H_2O_2-sensitive cysteine-containing peptides with a P value of < 0.05 and a difference in average % oxidation between H_2O_2-treated and control samples of > 7. Information about peptide sequences, Uniprot accession numbers, gene names and protein names are given for each peptide.

File Name: Supplementary Data 7
Description: Peptides identified in OxICAT experiments of *mia40-4int* and wild-type control samples. Reduced cysteine residues were labelled by heavy ICAT (^{13}C-ICAT). Reversibly oxidized cysteine residues were reduced by TCEP and labelled by light ICAT (^{12}C-ICAT). ICAT-labelled peptides were enriched by streptavidin affinity chromatography and analysed by LC-MS/MS. Mass spectrometric raw data were processed by MaxQuant (v.1.4.1.2) for peptide identification. For selected peptides, MS1 ion chromatograms were extracted using Skyline (v.2.5.0). Integrated peak areas of the "heavy" (^{13}C-ICAT-labelled) and "light" (^{12}C-ICAT-labelled) version of the peptide ions were exported and the proportion of reversibly oxidized (^{12}C-ICAT labelled) cysteine residues (% oxidation) was calculated. The table contains all cysteine-containing peptide sequences identified with posterior error probability (PEP) < 0.01 and intensity > 0 and all non-cysteine containing peptide sequences identified with PEP < 0.01. Reverse entries were removed.
