# Supplementary material for: Quantitative proteomics identifies redox switches for global translation modulation by mitochondrially produced reactive oxygen species
Source: Nat Commun. 2018 Jan 22;9:324. doi: 10.1038/s41467-017-02694-8 (PMC5778013; doi:10.1038/s41467-017-02694-8)
Supplement: Supplementary file 2 — Supplementary Information [file 41467_2017_2694_MOESM2_ESM.pdf]

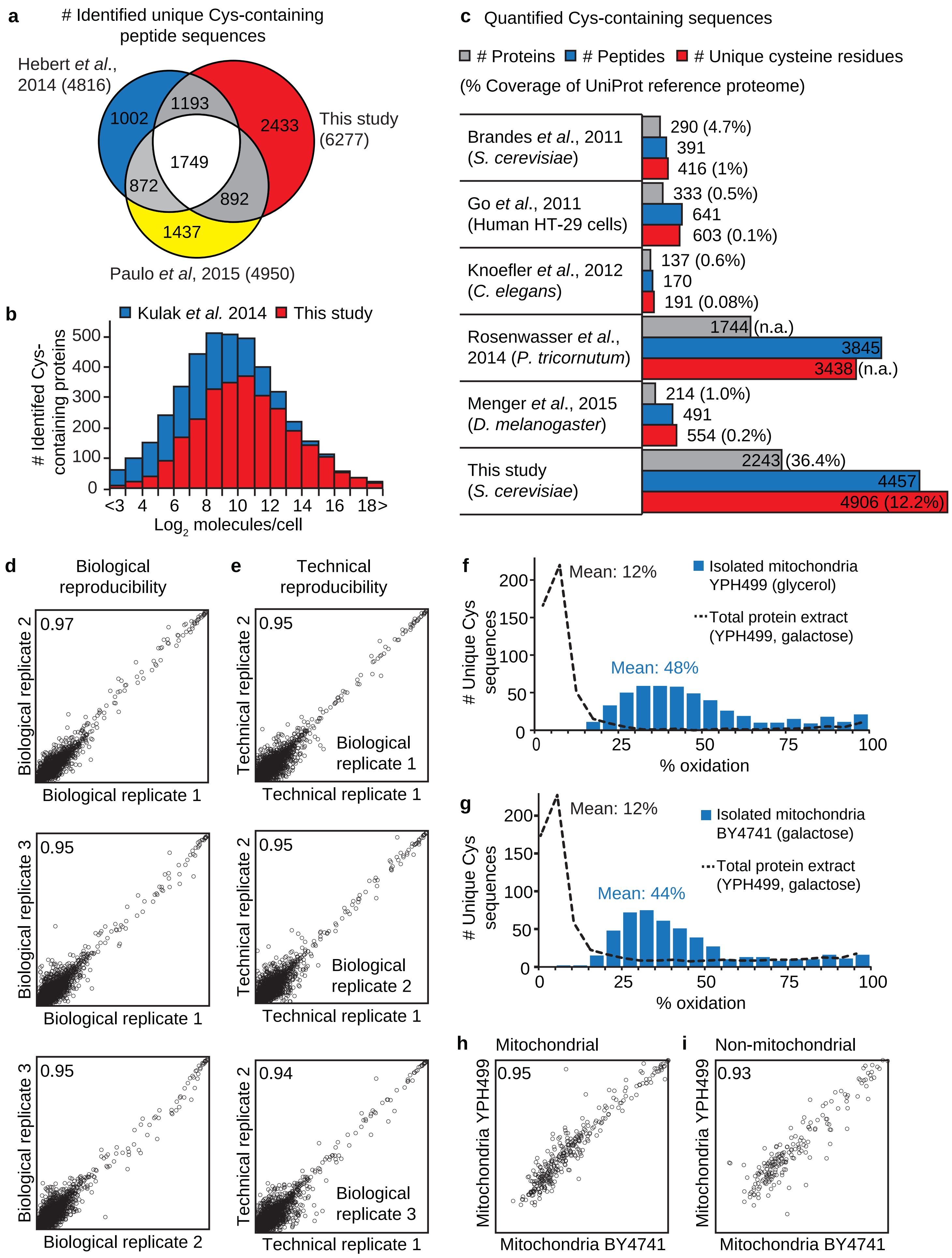

Supplementary Figure 1

Supplementary Figure 1 Performance of the methodology. **a** Comparison of the yeast thiol proteome dataset reported in this study with data from two recent shot-gun whole proteome studies of yeast[1,2]. In all three studies, a total of 10,469 unique cysteine-containing yeast peptides were identified with an overlap of 1,724 peptides (16.5%). Our dataset covers 61% and 52.5% of the unique cysteine-containing peptides reported by Hebert *et al.*[1] and Paulo *et al.*[2], respectively. 2,427 unique cysteine-containing peptides (23%) were uniquely identified in this study. **b** Distribution of cysteine-containing proteins identified in this study according to absolute abundance. Information about protein copy numbers was taken from previous work[3]. **c** Comparison of quantified cysteine-containing sequences in proteins reported in this study with previous OxICAT studies in eukaryotes. The % coverage of proteins and unique cysteine residues are given in brackets for the UniProt reference proteomes UP000002311 (*S. cerevisiae* strain ATCC 204508 / S288c), UP000005640 (*H. sapiens*), UP000001940 (*C. elegans*) and UP000000803 (*D. melanogaster*) respectively. n.a. not applicable. **d, e** Comparison of % oxidation values between biological (**d**) and technical (**e**) replicates of 4,457 cysteine-containing peptides quantified in at least two out of three biological replicates. **f**-**i** Redox state of proteins in mitochondrial fractions from cells of the *Saccharomyces cerevisiae* strains YPH499 and BY4741 grown in glycerol- and galactose-containing medium, respectively. **f**, **g** The distributions of the % oxidation values of 500 unique cysteine-containing peptides quantified in mitochondrial fractions from both yeast strains (blue bars) as well as in whole yeast cell extracts (black dashed line) directly frozen in 10% TCA are shown. **h, i** Comparison of the % oxidation values of 325 mitochondrial proteins (**h**) and 185 non-mitochondrial proteins (**i**) in the respective mitochondrial fractions. **d**, **e**, **h**, **i** Pearson correlation coefficients are shown in the upper left corner. Values on the x- and y-axis range from 0-100% oxidation.

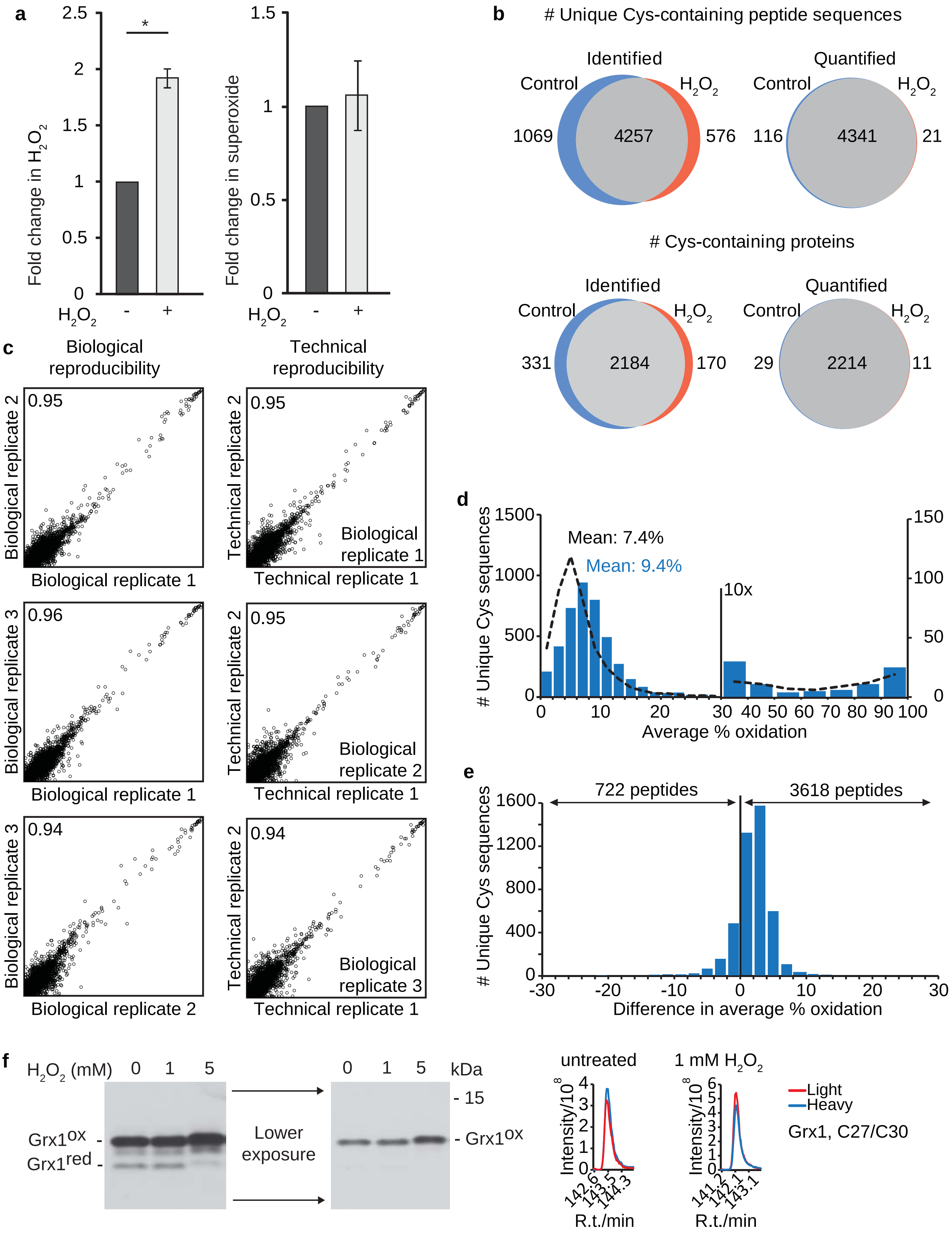

Supplementary Figure 2

Supplementary Figure 2 Large-scale analysis of the yeast redoxome following H_2O_2 treatment. **a** Wild-type cells were grown in fermentative medium at 28°C to mid-logarithmic growth phase. When indicated, cells were treated with 1 mM H_2O_2 for 30 min. Levels of H_2O_2 and superoxide were analysed. Mean +/- SEM, n=3, *P value < 0.05; two-sided, paired t-test. **b** Overlap of unique cysteine-containing peptides (top) and proteins (bottom) identified and quantified in at least two out of three biological replicates of untreated and H_2O_2-treated yeast cells. Using Skyline for accurate peptide quantification, the *in vivo* oxidation status of 4,341 unique cysteine-containing peptides in 2,214 proteins were determined both under basal and H_2O_2 conditions. **c** Average % oxidation values of 4,341 unique cysteine-containing peptides quantified in biological (left) and technical (right) replicates of H_2O_2-treated yeast cells. Pearson correlation coefficients are shown in the upper left corner. Values on the x- and y-axis range from 0-100% oxidation. **d** Average % oxidation status of 4,341 cysteine-containing peptides (blue bars) after treatment of wild-type yeast cells with 1 mM H_2O_2 for 30 min. For direct comparison, the distribution of the average % oxidation values of the peptides in untreated yeast cells is shown as dashed black line. **e** Shift in the *in vivo* thiol oxidation status upon H_2O_2 treatment. The differences in average % oxidation are shown for 4,341 unique cysteine-containing peptides from H_2O_2-treated and untreated (control) yeast cells. **f** Left. Total yeast cell extract was alkylated with 10 mM iodoacetamide, reduced with 50 mM TCEP and incubated with 10 mM AMS. Proteins were separated by SDS-PAGE, followed by immunodecoration with specific antibody. Right. Representative extracted ion chromatograms (XICs) of the triply charged peptide ion TYCPYCHAALNTLFEK+++ of Grx1 (containing cysteine residues 27 and 30) are shown for the untreated WT and following treatment with 1 mM H_2O_2. Red line, light ICAT-labelled peptide peak (oxidised); blue line, heavy ICAT-labelled peptide peak (reduced). R.t., retention time

a

b

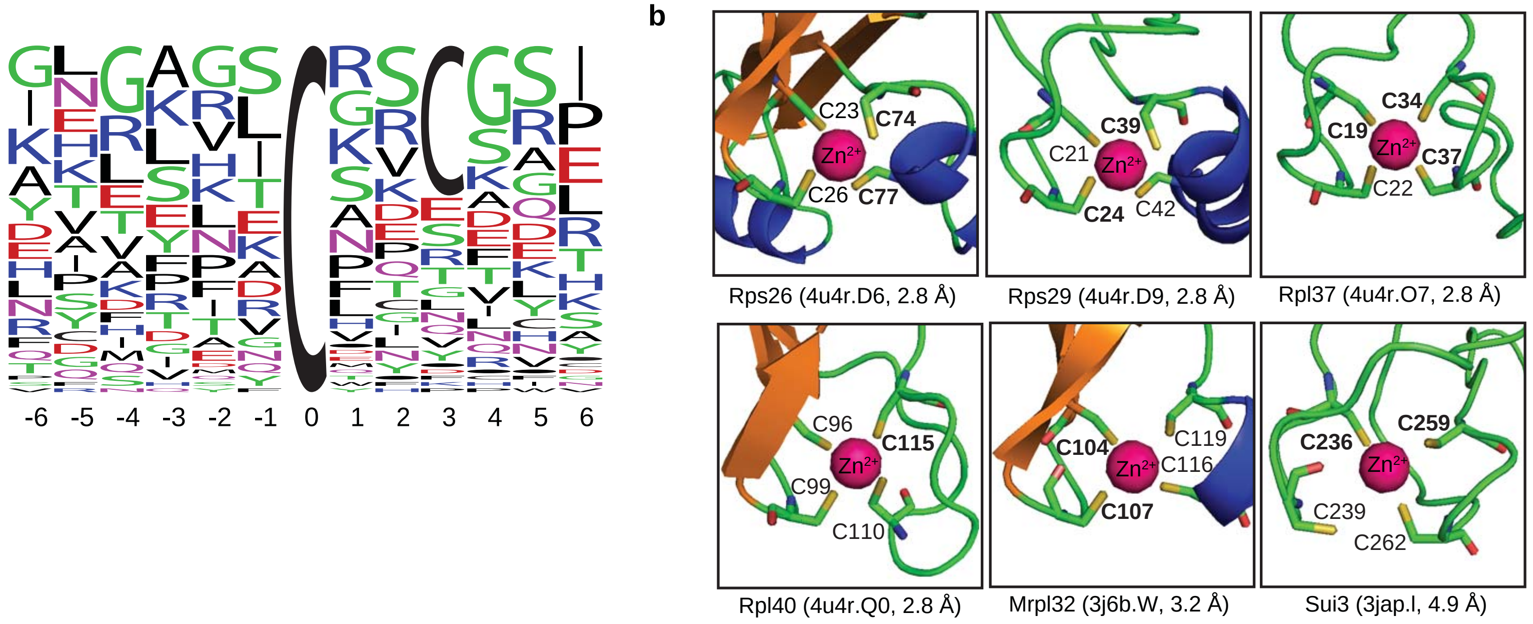

c

Rps26

```
                     CxxC                                                CxxC
P39939|YEAST 18 VKPVRCVNCSKSIPKDKAIKRMAIRNIVEAAAVRDLSEASVYPEYALPKTYNKLHYCVSCAIHAR 82
P62854|HUMAN 18 VQPIRCTNCARCVPKDKAIKKFVIRNIVEAAAVRDISEASVFDAYVLPKLYVKLHYCVSCAIHSK 82
P62855|MOUSE 18 VQPIRCTNCARCVPKDKAIKKFVIRNIVEAAAVRDISEASVFDAYVLPKLYVKLHYCVSCAIHSK 82
P13008|DROME 18 VKPVRCTNCARCVPKDKAIKKFVIRNIVEAAAVRDITEASIWDSYVLPKLYAKLHYCVSCAIHSK 82
O45499|CAEEL 18 VAFIRCTNCGRCCPKDKAIKKFVVRNIVEAAAVRDIGDASAYTQYALPKLYHKLHYCIACAIHSK 82
Q6P6E2|DANRE 18 VQPIRCTNCARCVPKDKAIKKFVIRNIVEAAAVRDISEASVFDSYVLPKLYVKLHYCVSCAIHSK 82
                *  :**.**.:. *******:..:***********: :** :  *.*** * *****::****::
```

Rps29

```
                     CxxC              CxxC
P41058|YEAST 16 KGSRQCRVCSSHTGLVRKYDLNICRQCFREKA 47
P62273|HUMAN 16 QGSRSCRVCSNRHGLIRKYGLNMCRQCFRQYA 47
P62274|MOUSE 16 QGSRSCRVCSNRHGLIRKYGLNMCRQCFRQYA 47
Q9VH69|DROME 16 QGSRCCRACSNRHGLIRKYGLNICRQCFREYA 47
P90983|CAEEL 16 PGSRSCRVCAGHHGLIRKYGLDLCRRCFREQA 47
Q6Q419|DANRE 16 QGSRSCRVCSNRHGLIRKYGLNMCRQCFRQYA 47
                ::****::* *** **.*: : **:*** *::
```

Rpl37

```
                     CxxC            CxxC
P49166|YEAST 14 KSHTLCNRCGRRSFHVQKKTCSSCGYPAA 42
P61927|HUMAN 14 KTHTLCRRCGSKAYHLQKSTCGKCGYPAK 42
Q9D823|MOUSE 14 KTHTLCRRCGSKAYHLQKSTCGKCGYPAK 42
M9PH59|DROME 14 KTHTLCRRCGRSSYHIQKSTCAQCGYPAA 42
P49622|CAEEL 14 KSHTLCKRCGKSSFHIQKKRCASCGYQDA 42
Q6IQJ7|DANRE 14 KTHTLCRRCGSKAYHLQKSTCGKCGYPAK 42
                *:****.***  ::*:**. *..***
```

Rpl40

```
                      CxxC          CxxxxC
P0CH09|YEAST 96 CDKSVCRKCYARLPPRATNCRKRKCGHTNQ 120
P62987|HUMAN 96 CDKMICRKCYARLHPRAVNCRKKKCGHTNN 120
P62984|MOUSE 96 CDKMICRKCYARLHPRAVNCRKKKCGHTNN 120
P18101|DROME 96 CDKMICRKCYARLHPRATNCRKKKCGHTNN 120
P49632|CAEEL 96 CDKQICRKCYARLPPRASNCRKKKCGHSSE 120
Q3B7P7|DANRE 96 CEKMICRKCYARLHPRAVNCRKKKCGHTNN 120
                *:* :******** *** ****:****:.:
```

Mrpl32

```
                       CxxC          CxxC
P25348|YEAST  99 HHLNKCPSCGHYKRANTLCMYCVGQIS 125
Q9BYC8|HUMAN 105 NNIDVCPECGHLKQKHVLCAYCYEKVC 131
Q9DCI9|MOUSE 104 NNIDICPECGHLKQKHVLCGYCYEKVR 130
Q9V9Z1|DROME  90 RNLRSCLQCGHDHEMGVLCPFCYQKVL 116
Q04907|CAEEL  94 DNLVTCPACSNIHPSDTICDACYAKVH 120
Q0P472|DANRE  92 YNFEPCLECGNMKLKHTLCGFCYEKIS 118
                  .:  *  *.. :   .:*  *  ::
```

Sui3

```
                       CxxC                    CxxC
P09064|YEAST 231 LEYVTCKTCKSINTELKREQSNRLFFMVCKSCGSTRS 268
P20042|HUMAN 276 KEYVTCHTCRSPDTILQK--DTRLYFLQCETCHSRCS 313
Q99L45|MOUSE 268 KEYVTCHTCRSPDTILQK--DTRLYFLQCETCHSRCS 305
P41375|DROME 255 KEYVTCHTCRSPETILQK--DTRLFFLQCESCGSRCS 292
Q21230|CAEEL 188 KEYVMCHTCKSPETQLTK--DTRLFFLQCTNCGSRCS 225
Q6NY78|DANRE 270 KEYVTCHTCRSPDTILQK--DTRLYFLQCETCHSRCS 307
                  *** *:**:* :* * :  ..**:*: * .* *  *
```

Supplementary Figure 3

Supplementary Figure 3 ROS-sensitive cysteine residues are part of conserved sequence motifs in proteins involved in translation. **a** Graphical representation of amino acid frequency within 54 significantly H_2O_2-sensitive sequences covering six amino acids upstream and downstream of the first quantified cysteine residue. The plot was generated using Weblogo 2.8.2[4]. **b** Comparison of structural properties of redox-sensitive thiols that lie within a $CX_2C\text{-}X_{(9\text{-}47)}\text{-}CX_{(2\text{-}4)}C$ motif. Cysteine residues were mapped onto published crystal structures[5-7]. Respective protein database (pdb) accession numbers, chain names and resolution are shown in brackets for each protein. H_2O_2-sensitive cysteine residues are labelled in bold. Structures were visualized using PyMOL (The PyMOL Molecular Graphics System, Version 1.3 Schrödinger, LLC). **c** Homologues to the indicated yeast proteins were identified by BLAST searches in UniProt against UniProtKB sub-databases Vertebrates (*Homo sapiens* (HUMAN), *Mus musculus* (MOUSE), *Danio rerio* (DANRE), Arthropoda (*Drosophila melanogaster*, DROME) and Nematoda (*Caenorhabditis elegans*, (CAEEL) respectively. Multiple sequence alignments were constructed using Clustal O (1.2.1)[8]. In each case, the $CX_2C\text{-}X_{(9\text{-}47)}\text{-}CX_{(2\text{-}4)}C$ motif and five adjacent N- and C-terminal amino acid residues of the alignment are shown. ROS-sensitive cysteine residues identified and quantified in this work are shown in bold in the yeast sequence and conserved cysteine residues are highlighted in yellow. ROS, reactive oxygen species

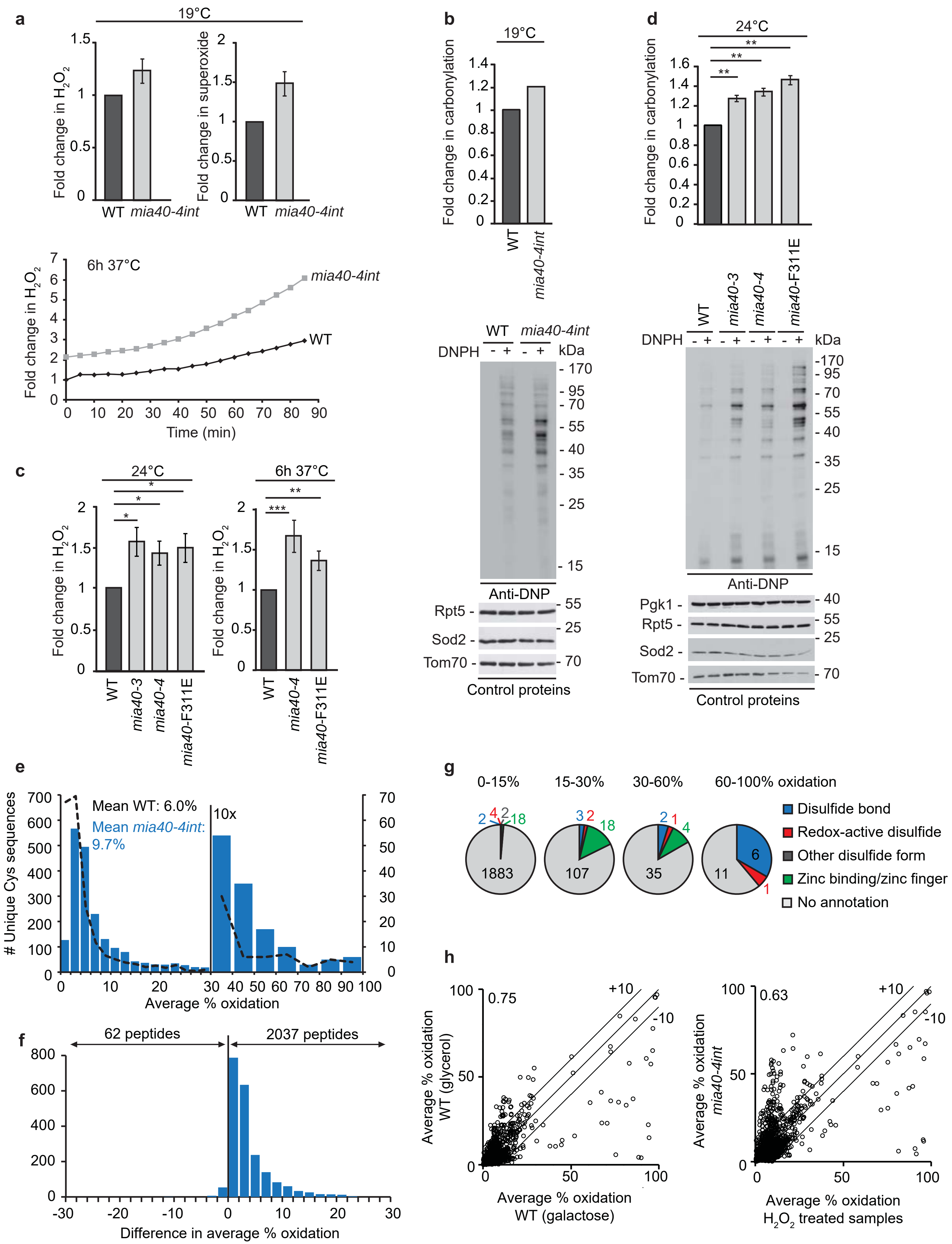

Supplementary Figure 4

Supplementary Figure 4 Analysis of oxidative stress in yeast *mia40* mutants. **a** Top. Endogenous levels of H_2O_2 or superoxide in *mia40-4int* and wild-type strains at 19°C. Mean +/- SEM, n=3. Bottom. The *mia40-4int* and wild-type strains were grown in respiratory medium at 19°C and shifted to 37°C for 6 h. After 40 min incubation with the dye CM-H_2DCFDA, the level of endogenous H_2O_2 was measured every 5 min for 85 min. Mean, n=4. **b** Top. Carbonylation of proteins in *mia40-4int* and wild-type strains grown at 19°C was quantified by spectrophotometry. Mean, n=2. Bottom. Protein carbonylation was analysed by immunoblotting with anti-DNP antibody. Equal protein loading was controlled by immunoblotting using specific antibodies. **c** Endogenous levels of H_2O_2 in *mia40* mutants and the corresponding wild-type strain grown in respiratory medium at 24°C or shifted to 37°C for 6 h. Left panel: mean +/- SEM, n=5. *, P value < 0.05. Right panel: mean +/- SEM, n=6. **, P value < 0.02; ***, P value < 0.01. P values were calculated using a two-sided, paired t-test. **d** Top. Protein carbonylation in *mia40* mutants and the corresponding wild-type strain grown at 24°C was quantified by spectrophotometry. Mean +/- SEM, n=4. **, P value < 0.02; two-sided, paired t-test. Bottom. Protein carbonylation was analysed by immunoblotting with anti-DNP antibody. Equal protein loading was controlled by immunoblotting using specific antibodies. DNPH, 2,4-dinitrophenylhydrazine. **e** Average % oxidation status of 2,099 cysteine-containing peptides quantified in at least three out of four biological replicates in both *mia40-4int* (blue bars) and in wild-type cells (dashed black line). **f** *mia40-4int*-dependent changes in the *in vivo* oxidation status of protein thiols. The differences in average % oxidation are shown for 2,099 unique cysteine-containing peptides from *mia40-4int* and wild-type (control) yeast cells. **g** Average % oxidation values of 2,099 unique cysteine-containing peptides quantified in at least three out of four biological replicates in the wild-type control were classified into four oxidation groups as indicated. Frequency of disulfide bond and zinc binding and/or zinc finger annotations within the different oxidation groups is shown. Other disulfide forms include transient disulfide bonds and those in linked or nuclear-retained form. **h** Comparison of the thiol oxidation status of 1,785 unique cysteine-containing peptides quantified in both datasets. Pearson correlation coefficients are shown in the upper left corner. Diagonal is shown and additional lines indicate an increase or decrease in average % oxidation by 10 as

indicated. Left. Comparison of average % oxidation values between untreated control cells (wild-type, grown in galactose) and wild-type control for *mia40-4int* (grown in glycerol). Right. Comparison of average % oxidation values between H_2O_2 treated cells and *mia40-4int*. WT, wild-type

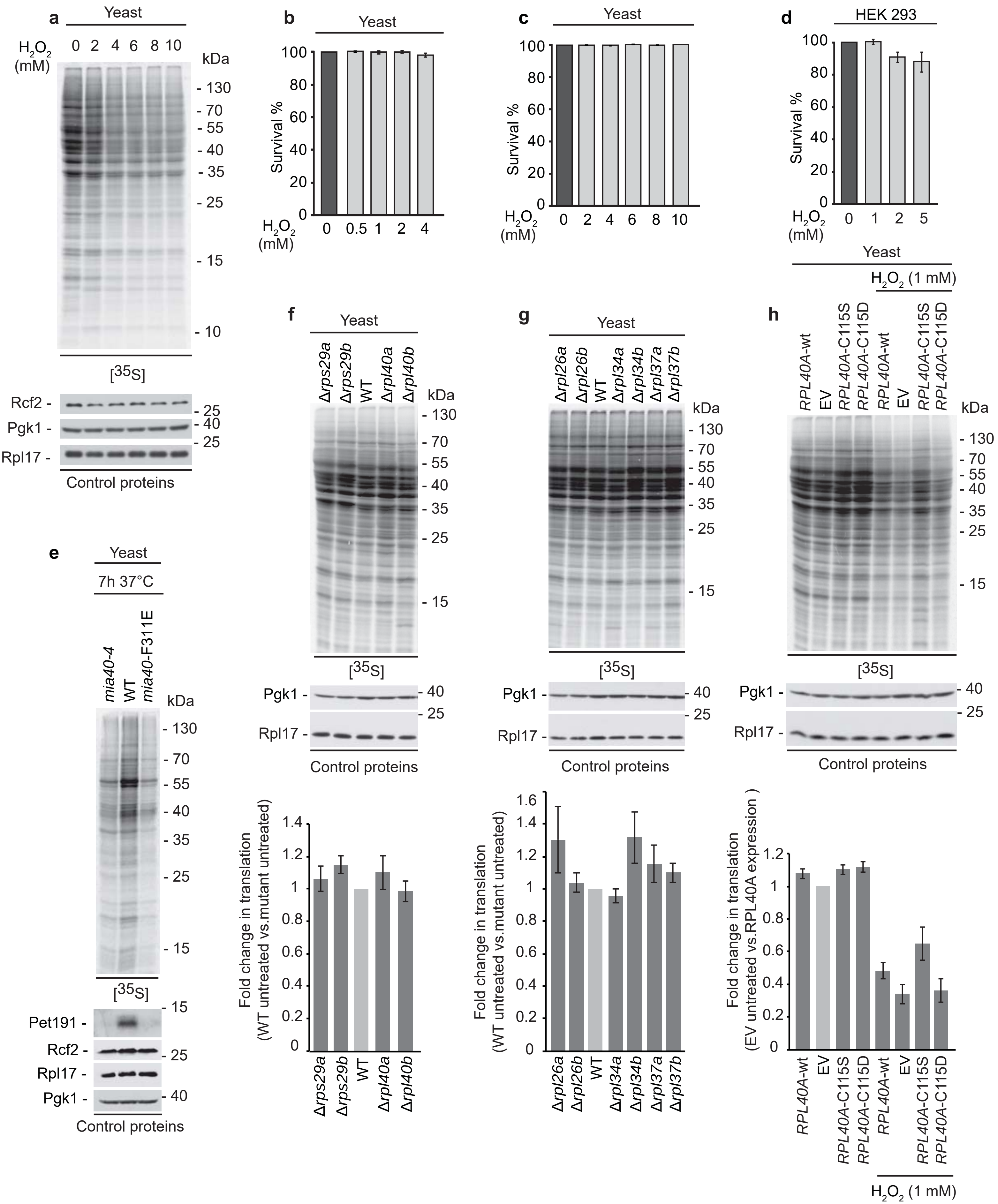

Supplementary Figure 5

Supplementary Figure 5 ROS production affects translation **a, e-h** Incorporation of [^{35}S]-labelled amino acids. Total cell extracts were separated by SDS-PAGE and analysed by autoradiography or immunodecorated with specific antibodies. **a** Wild-type (YPH499) yeast cells were grown on fermentative medium and treated for 30 min with H_2O_2 as indicated. Incorporation of [^{35}S]-labelled amino acids was done at the same time as treatment with H_2O_2. **b, c** Survival of wild-type yeast (BY4741 (b), YPH499 (c)) treated with different concentrations of H_2O_2 for 30 min. Mean +/- SEM, n=3. **d** Survival of HEK 293 cells treated for 2 h with H_2O_2 as indicated. Mean +/- SEM, n=3. **e** The *mia40-4, mia40*-F311E mutants and the corresponding wild-type strain were grown on respiratory medium at 19°C to logarithmic growth phase and shifted for 7 h to restrictive temperature (37°C). One hour prior harvesting, cells were incubated with [^{35}S]-labelled amino acids. **f, g** Wild-type (BY4741) yeast cells and cells with deletions of ribosomal genes were grown on fermentative medium (upper panel). Quantification of protein synthesis. Mean +/- SEM, n=3 (lower panel). **h**, Wild-type (BY4741) yeast cells were transformed with empty vector (EV) or vector constitutively expressing wild-type *RPL40A* protein (*RPL40A*-wt) or mutant forms of *RPL40A* (*RPL40A*-C115S, *RPL40A*-C115D). Yeast cells were grown on fermentative medium and treated or not with H_2O_2 for 30 min (upper panel). Quantification of protein synthesis. Mean +/- SEM, n=3 (lower panel). WT, wild-type

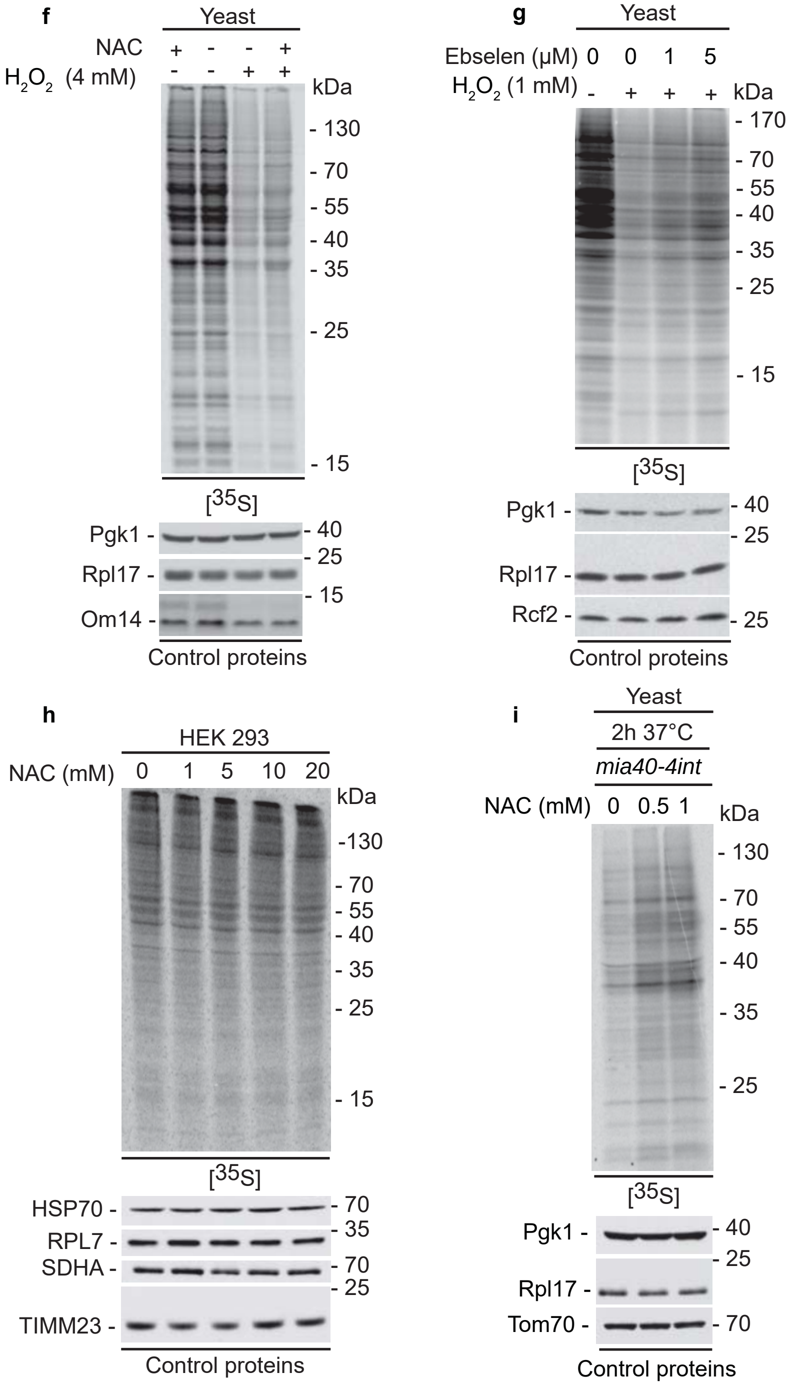

Supplementary Figure 6

Supplementary Figure 6 Protein synthesis defect is reversible **a** Wild-type (YPH499) yeast cells were grown on fermentative medium supplemented with or without 100 mM N-acetylcysteine for 4 h as indicated. Cells were washed and resuspended in fresh medium without N-acetylcysteine, but with H_2O_2 where indicated and further incubated for 30 min. **b** Wild-type (BY4741) yeast cells were treated for 1 h with different concentrations of ebselen as indicated. 30 min prior harvesting, cells were treated with H_2O_2. **c** Treatment of HEK 293 cells for 2 h with different concentrations of N-acetylcysteine. **d** *Mia40-4int* cells were grown on respiratory medium at 19°C and shifted for 2 h to restrictive temperature (37°C). During growth at restrictive temperature, yeast cells were treated with N-acetylcysteine at indicated concentrations. NAC, N-acetylcysteine; WT, wild-type

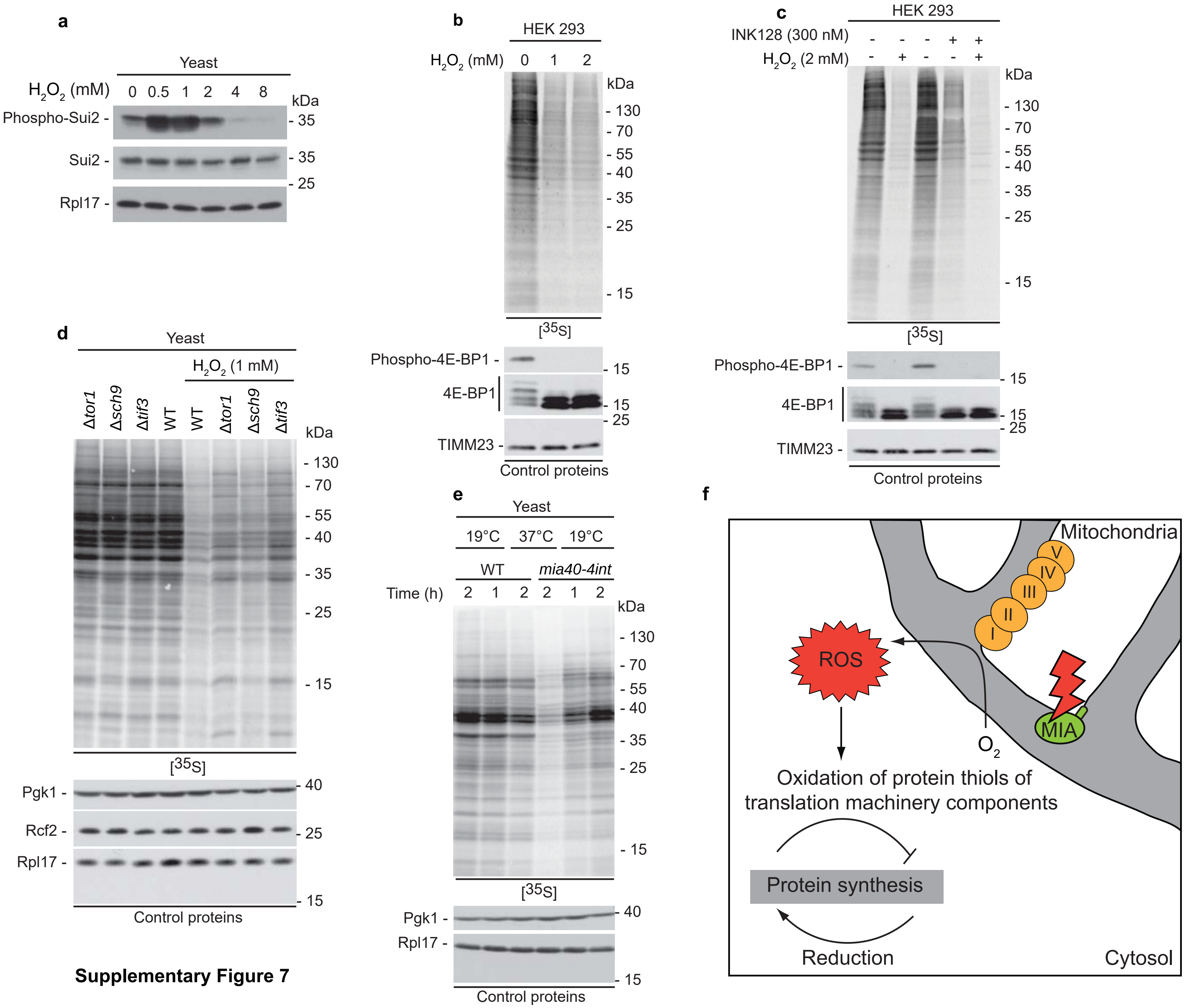

Supplementary Figure 7

Supplementary Figure 7 Mechanisms involved in translation attenuation upon H_2O_2 treatment. **a** Wild-type (BY4741) yeast cells were treated for 30 min with different concentrations of H_2O_2 as indicated. Total protein extracts were analysed by SDS-PAGE followed by immunoblotting. **b-e** Incorporation of [^{35}S]-labelled amino acids into newly synthesized proteins. Total cell extracts were separated by SDS-PAGE and analysed by autoradiography or immunodecorated with specific antibodies. Antibody against phospho-4E-BP1 (Ser65) was used as readout for mTOR kinase activity (b, c). **b** HEK 293 cells were treated for 2 h with H_2O_2 as indicated. **c** HEK 293 cells were treated for 3 h with INK128 (mTOR kinase inhibitor) where indicated. 2 h prior harvesting, cells were treated with H_2O_2 where indicated. **d** Wild-type (BY4741) and deletion yeast strains were treated for 30 min with or without H_2O_2. **e** *Mia40-4int* and wild-type cells were grown on respiratory medium at 19°C and shifted for 2 h to restrictive temperature (37°C). Afterwards cells were shifted back to permissive temperature (19°C). Samples for SDS-page analysis were taken at indicated time points. **f** Model for the redox regulation of cytoplasmic translation by mitochondrially produced reactive oxygen species. ROS, reactive oxygen species; MIA, mitochondrial intermembrane space assembly; I-V, respiratory chain complex I-V; WT, wild-type.

Figure 2

Fig. 2b

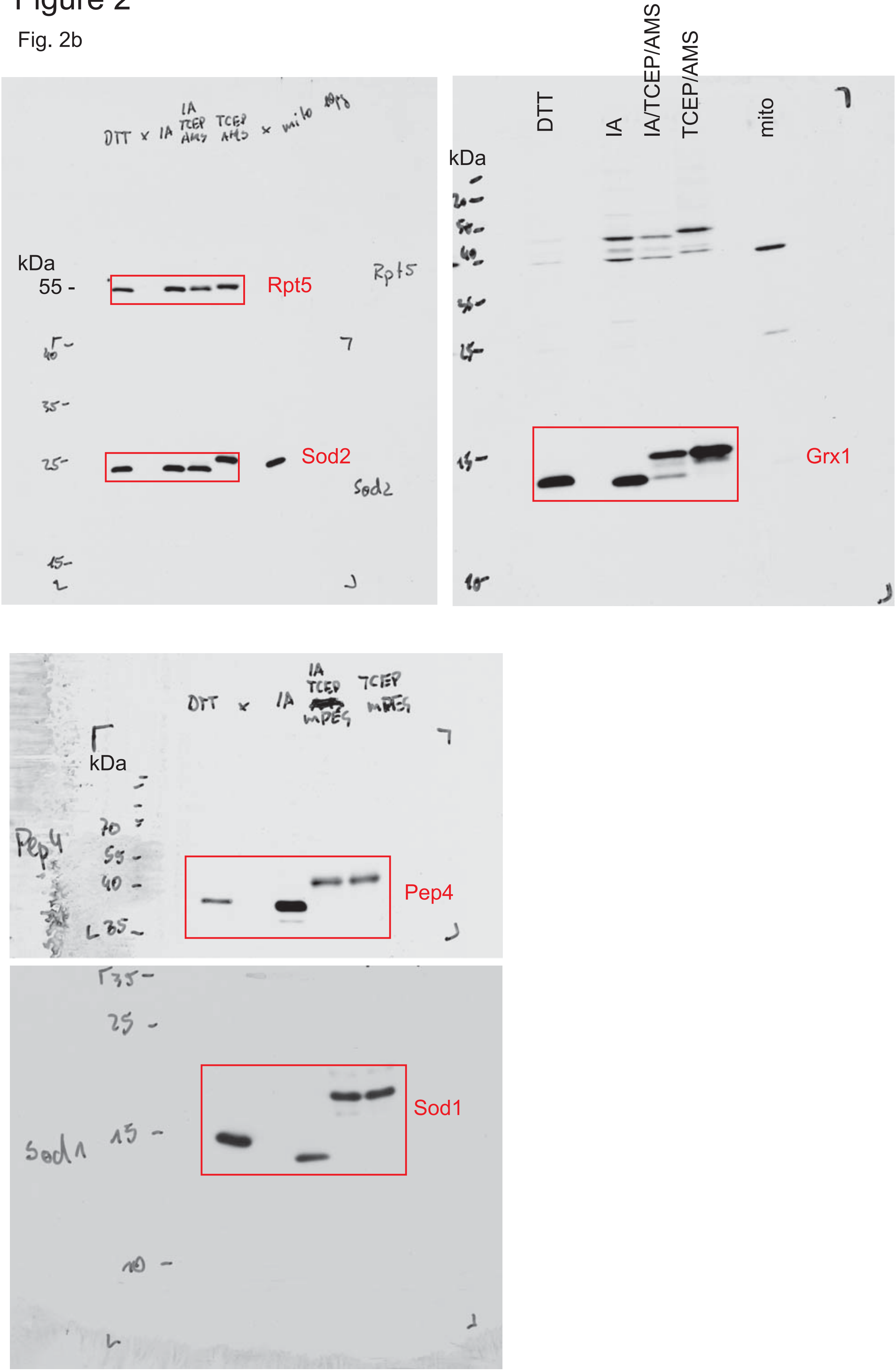

After protein transfer membranes were cut and their fragments were incubated with antibodies against indicated proteins.

Figure 3

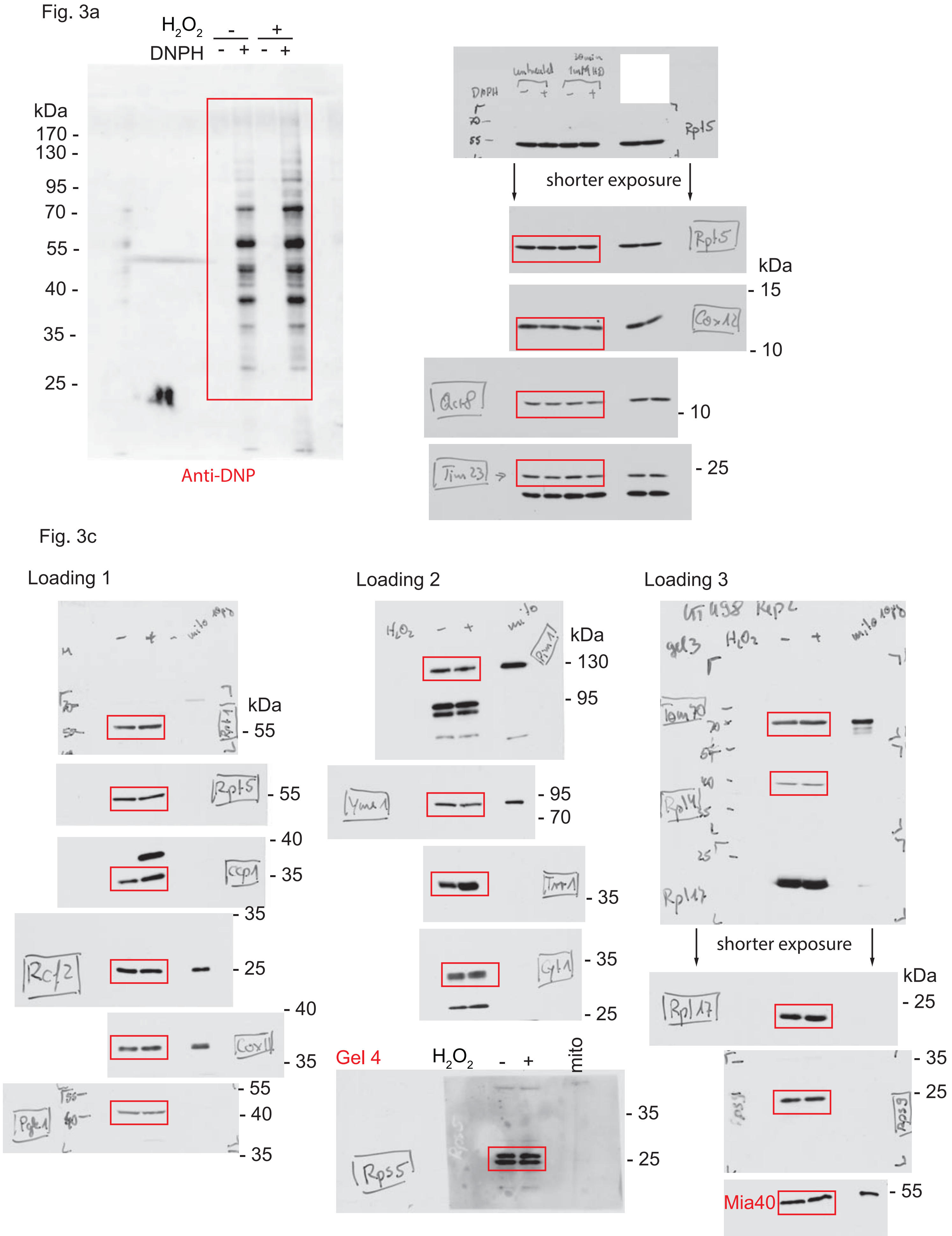

After protein transfer membranes were cut and their fragments were incubated with antibodies against indicated proteins.

Figure 4

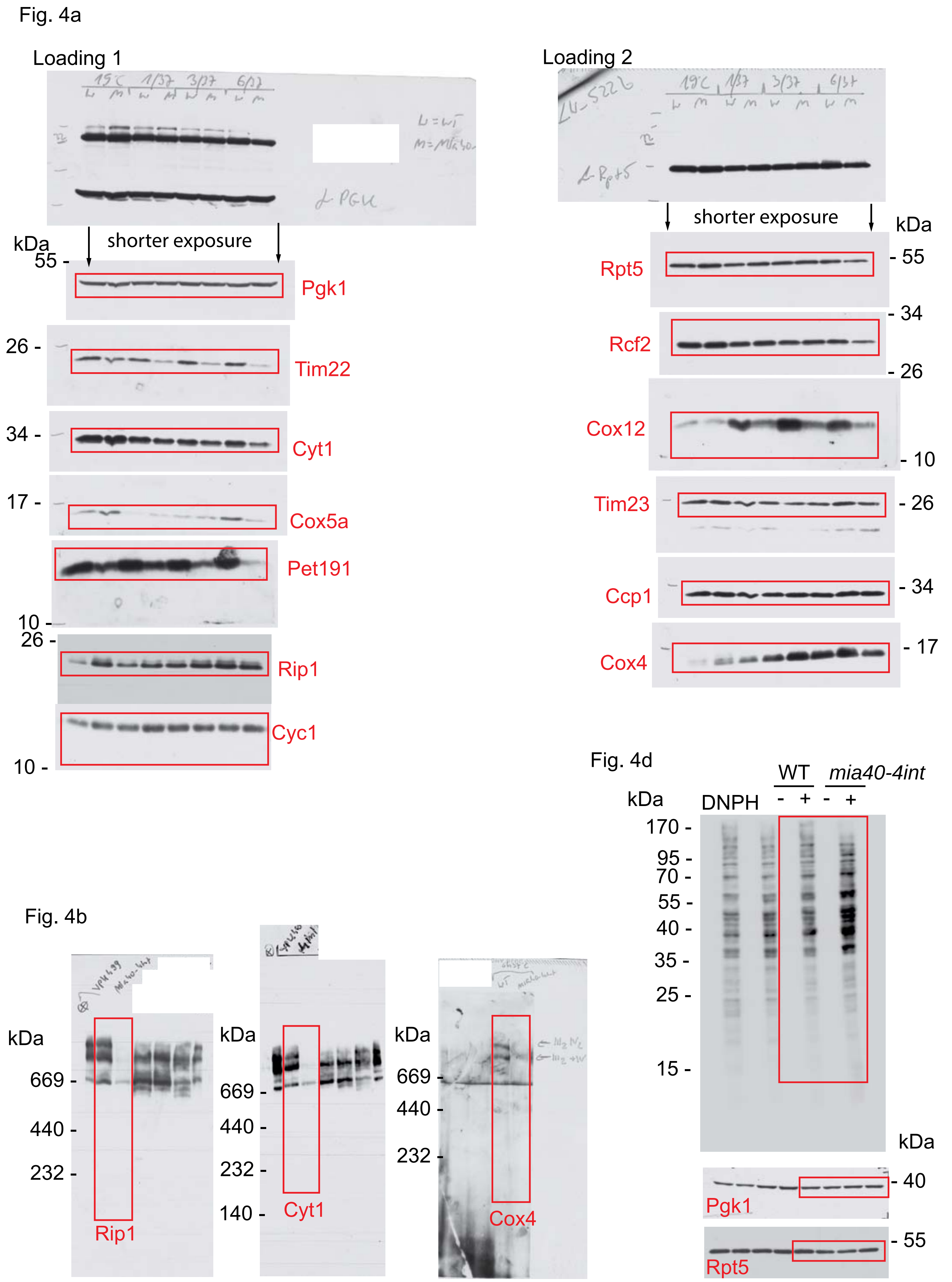

After protein transfer membranes were cut and their fragments were incubated with antibodies against indicated proteins.

Figure 6

Fig. 6a and Supplementary Fig. 6a

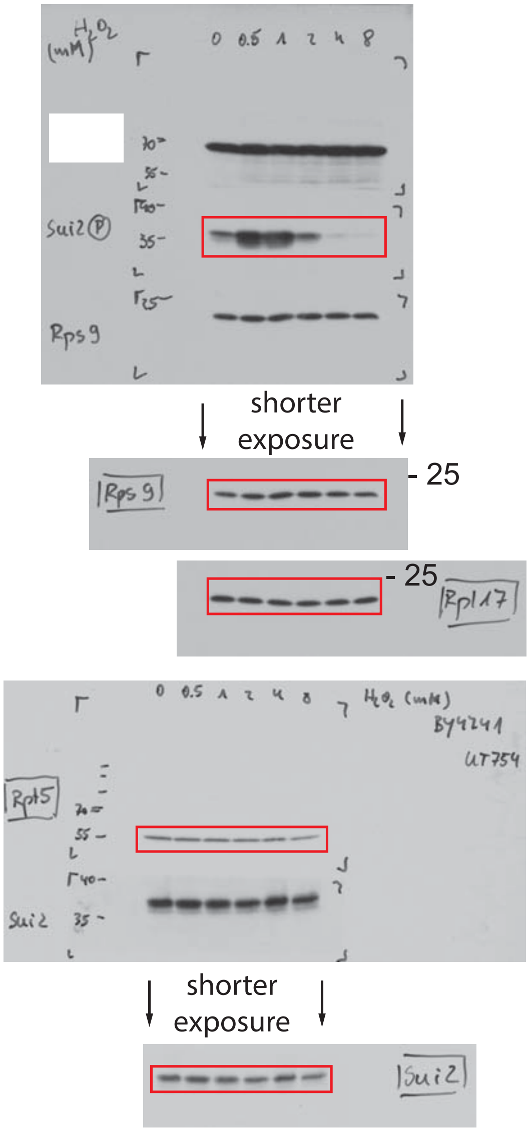

Fig. 6a

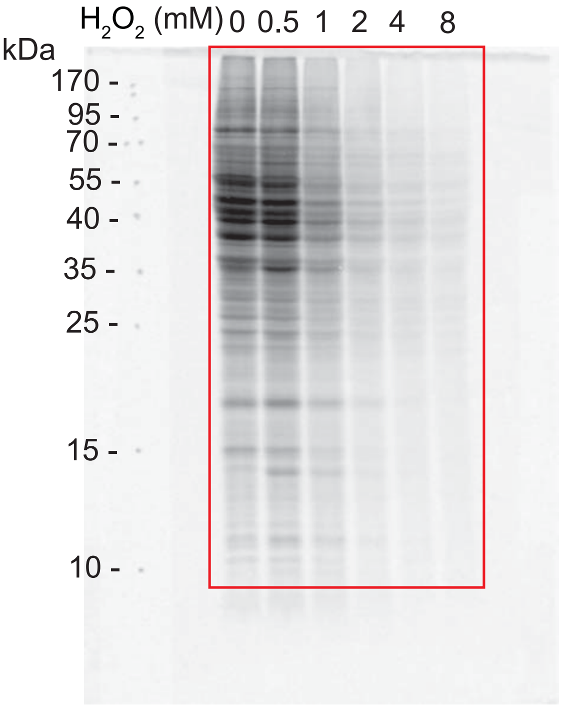

Fig. 6b

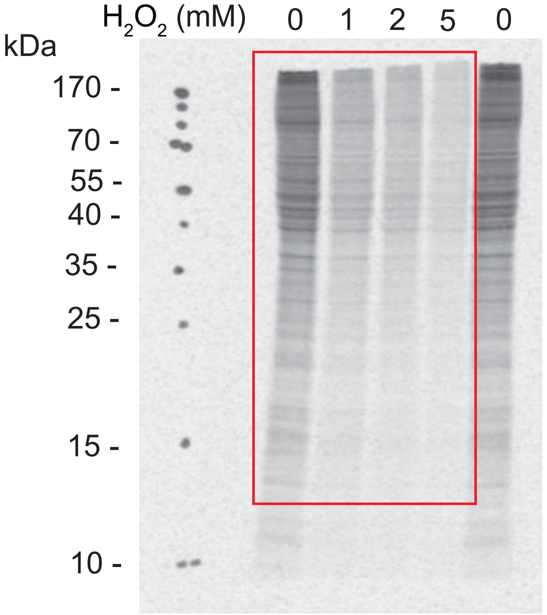

Fig. 6b

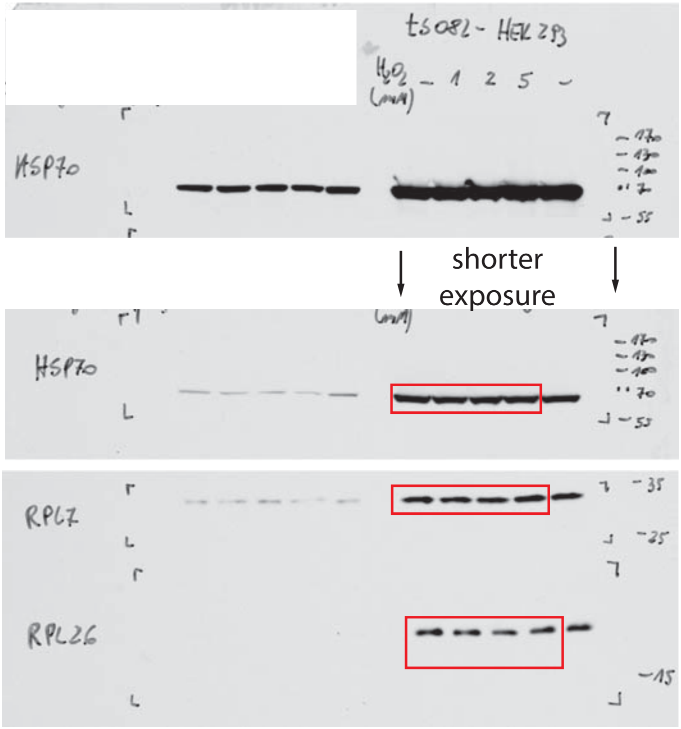

After protein transfer membranes were cut and their fragments were incubated with antibodies against indicated proteins.

Figure 6

Fig. 6c

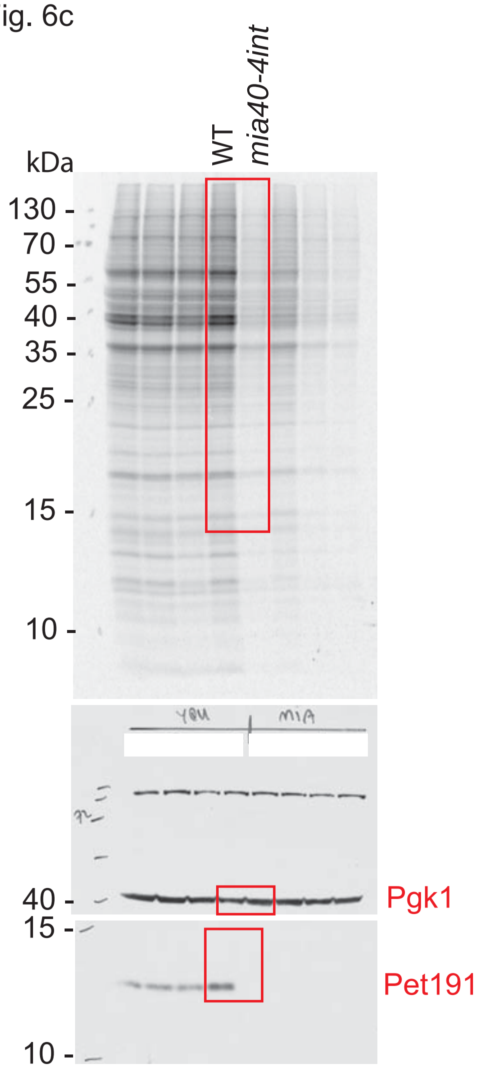

Fig. 6d and Supplementary Fig. 5f

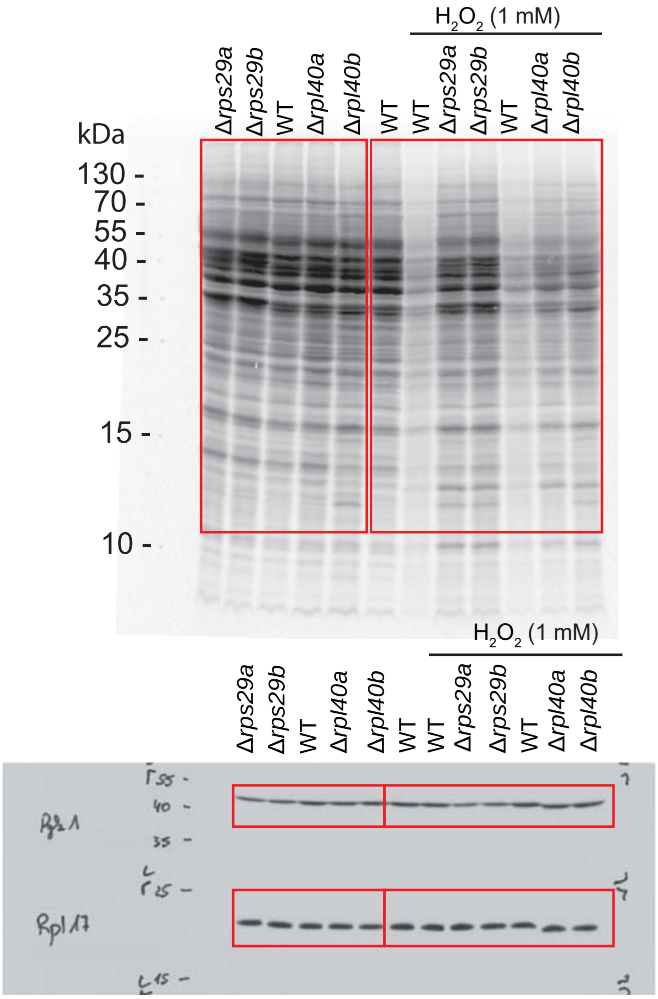

Fig. 6e and Supplementary Fig. 5g

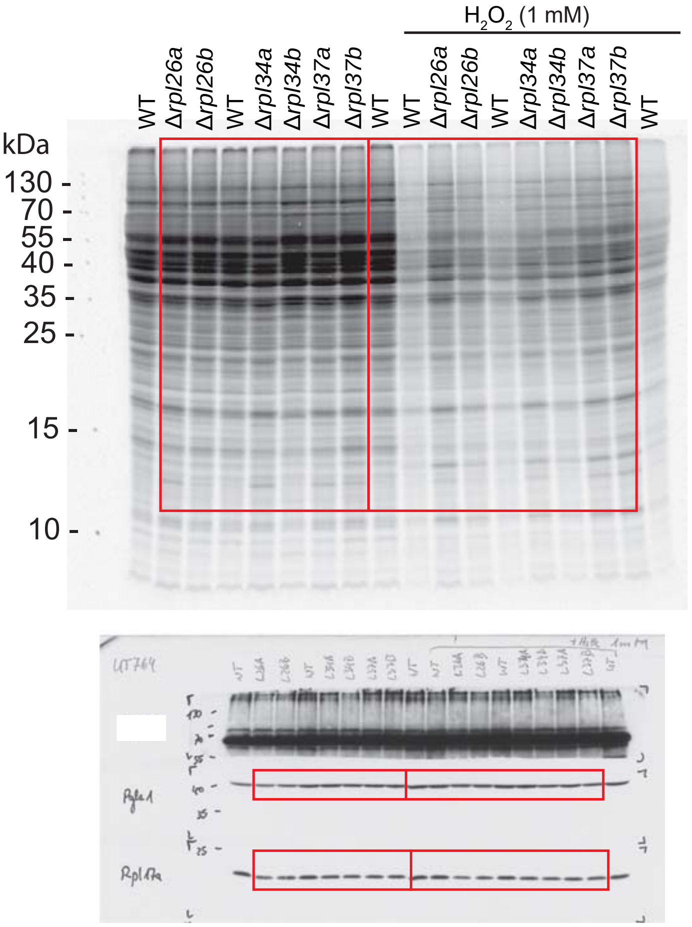

After protein transfer membranes were cut and their fragments were incubated with antibodies against indicated proteins.

Figure 7

Fig. 7a

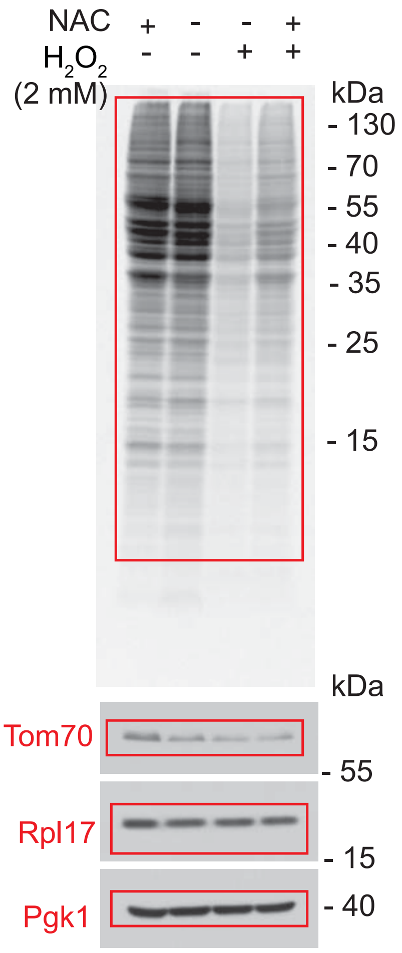

Fig. 7b

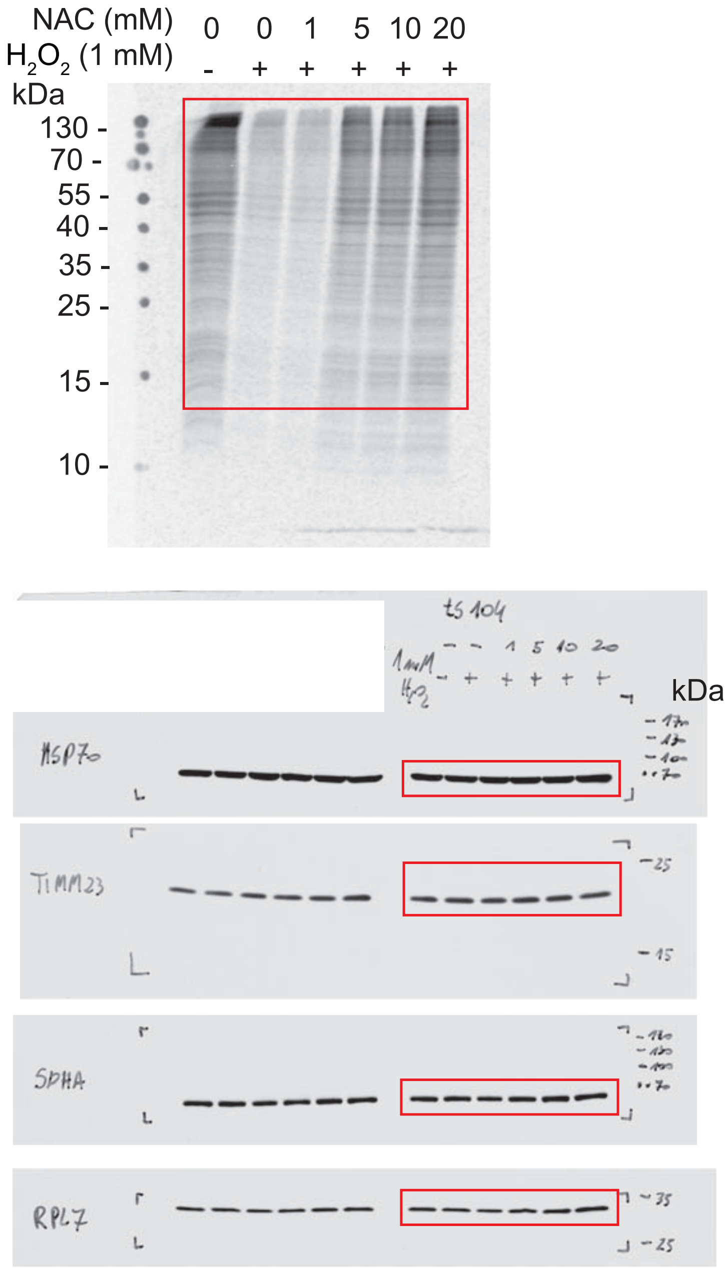

After protein transfer membranes were cut and their fragments were incubated with antibodies against indicated proteins.

Figure 7

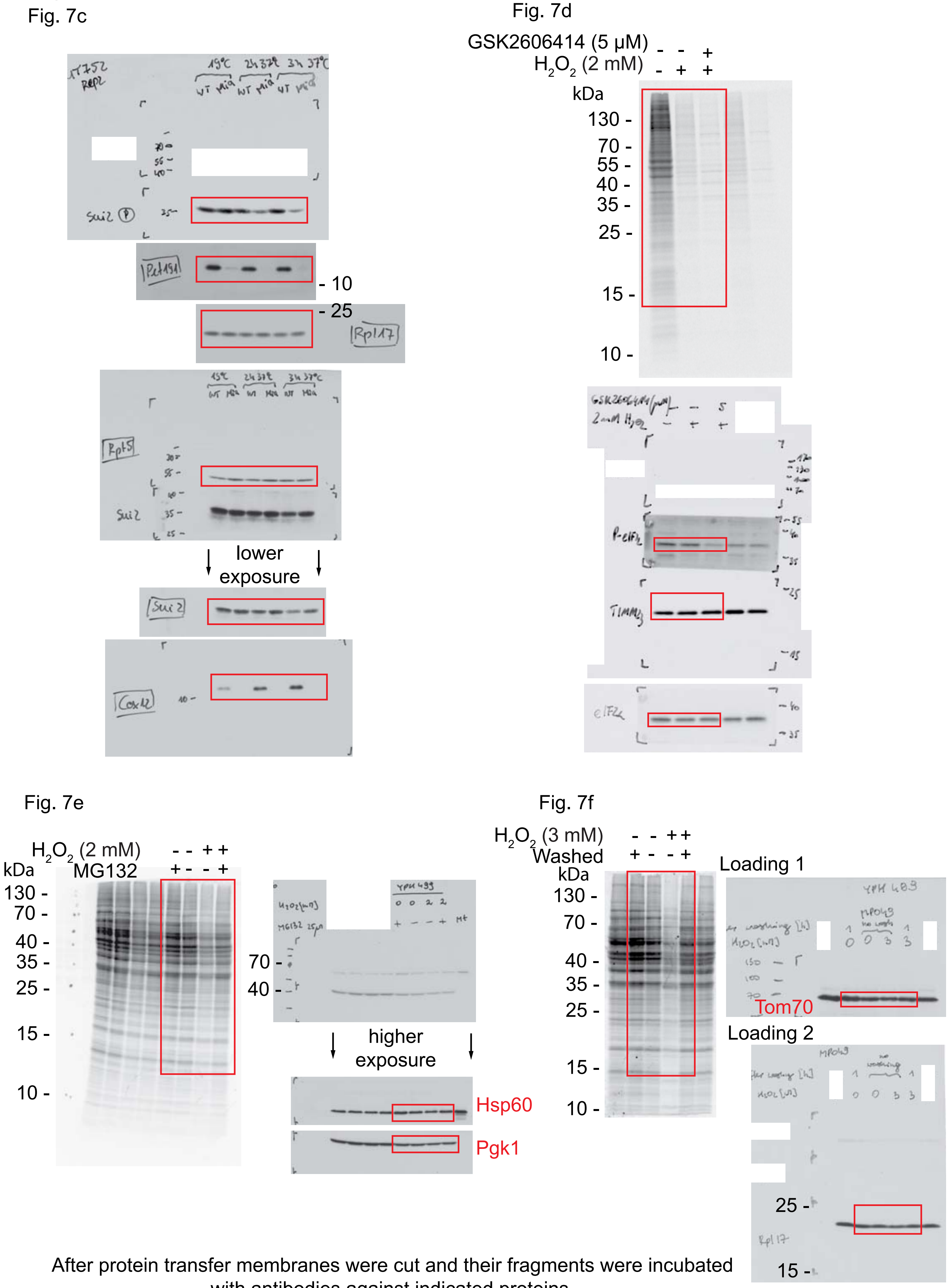

After protein transfer membranes were cut and their fragments were incubated with antibodies against indicated proteins.

Supplementary Figure 2

Supplementary Fig. 2f

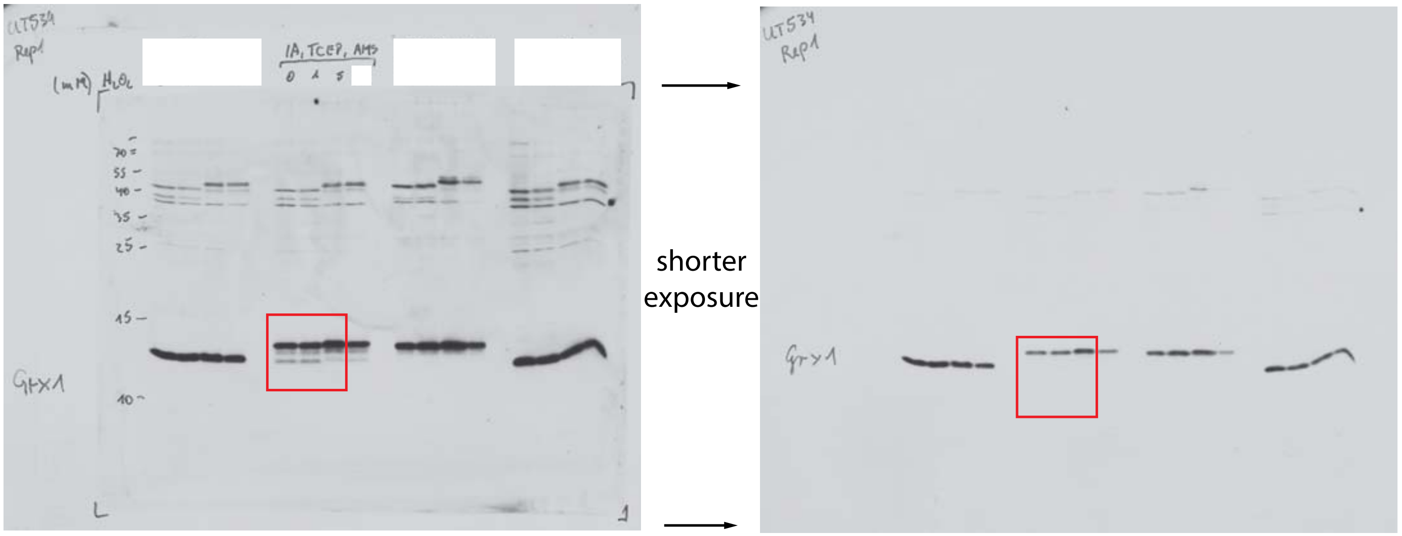

Supplementary Figure 4

Supplementary Fig. 4b

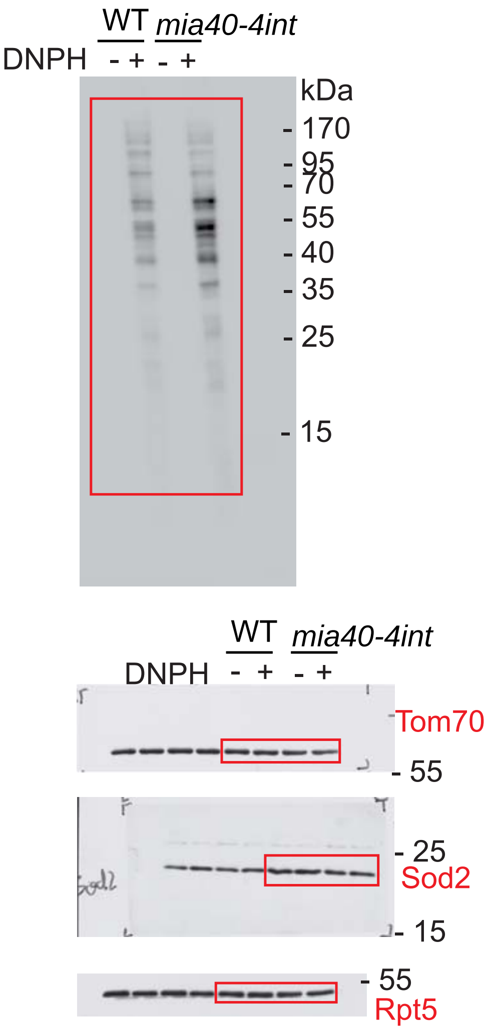

Supplementary Fig. 4d

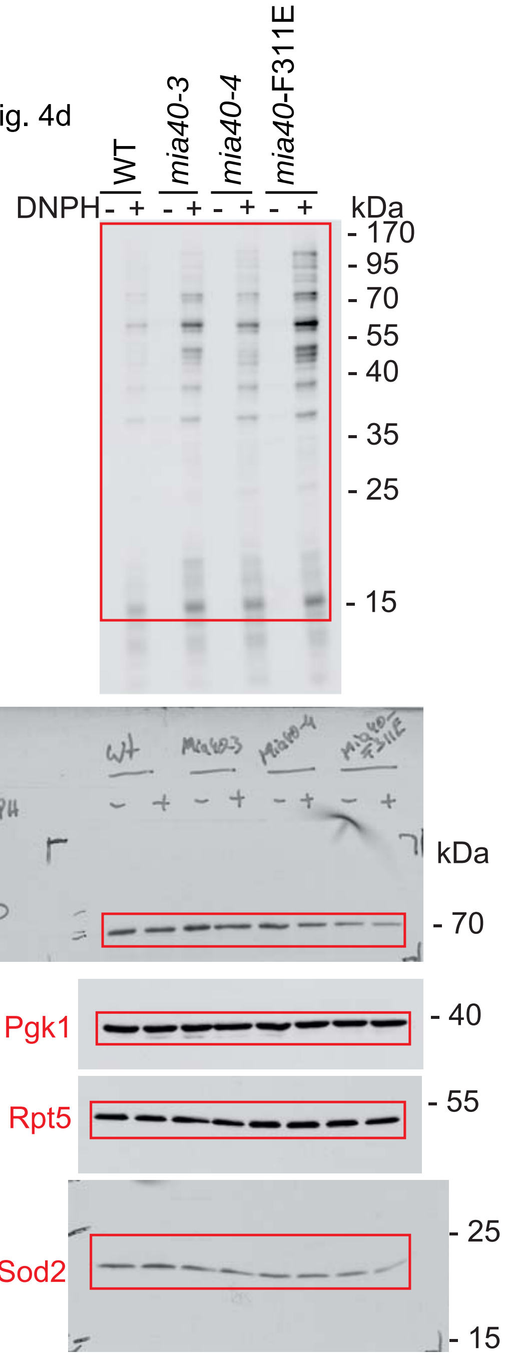

After protein transfer membranes were cut and their fragments were incubated with antibodies against indicated proteins.

Supplementary Figure 5

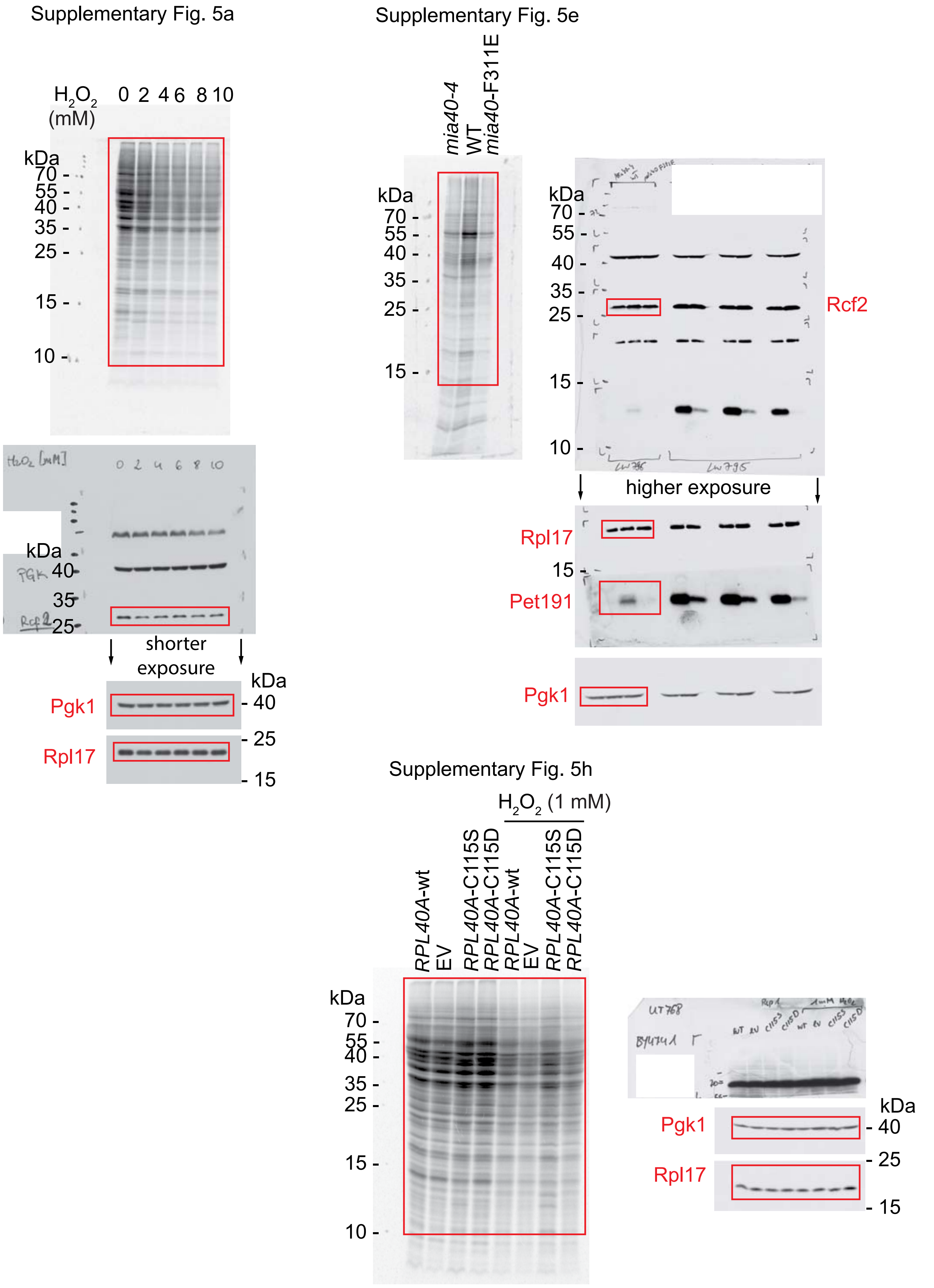

After protein transfer membranes were cut and their fragments were incubated with antibodies against indicated proteins.

Supplementary Figure 6

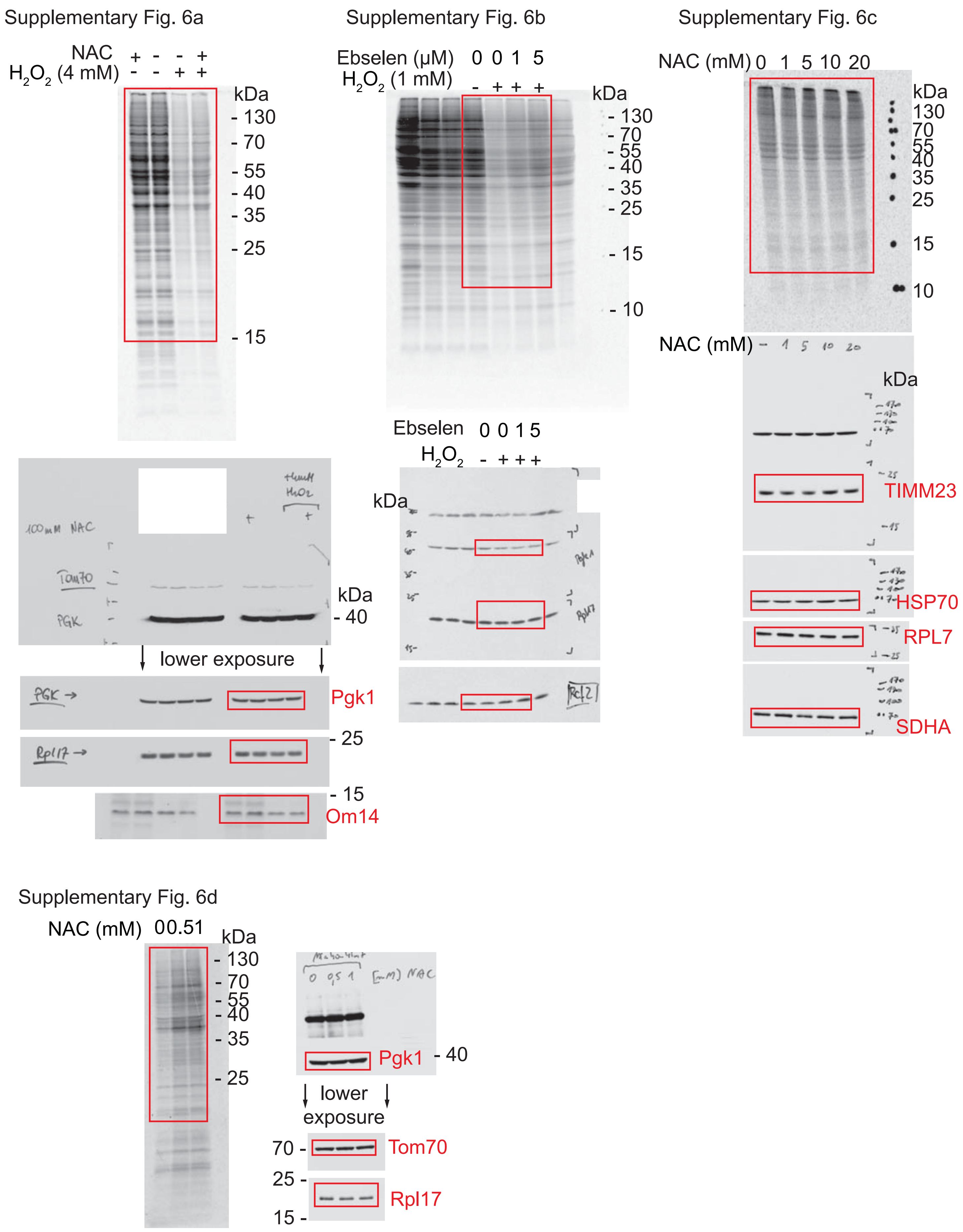

After protein transfer membranes were cut and their fragments were incubated with antibodies against indicated proteins.

Supplementary Figure 7

Supplementary Fig. 7b

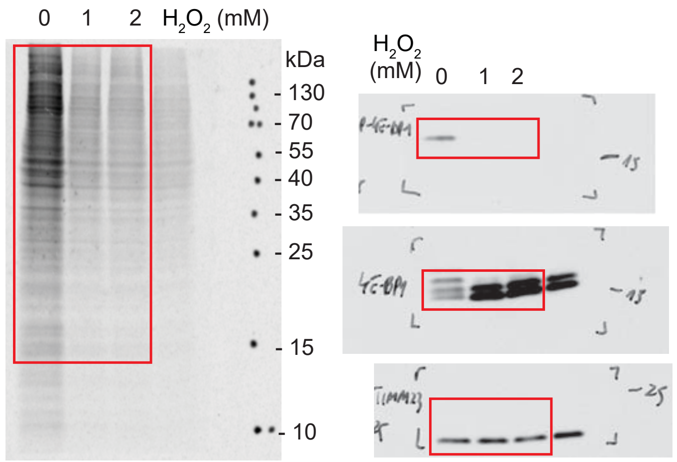

Supplementary Fig. 7c

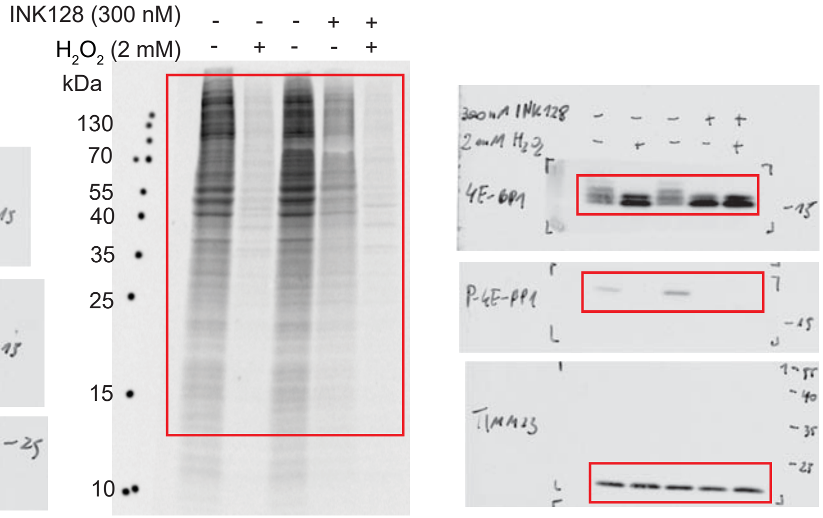

Supplementary Fig. 7d

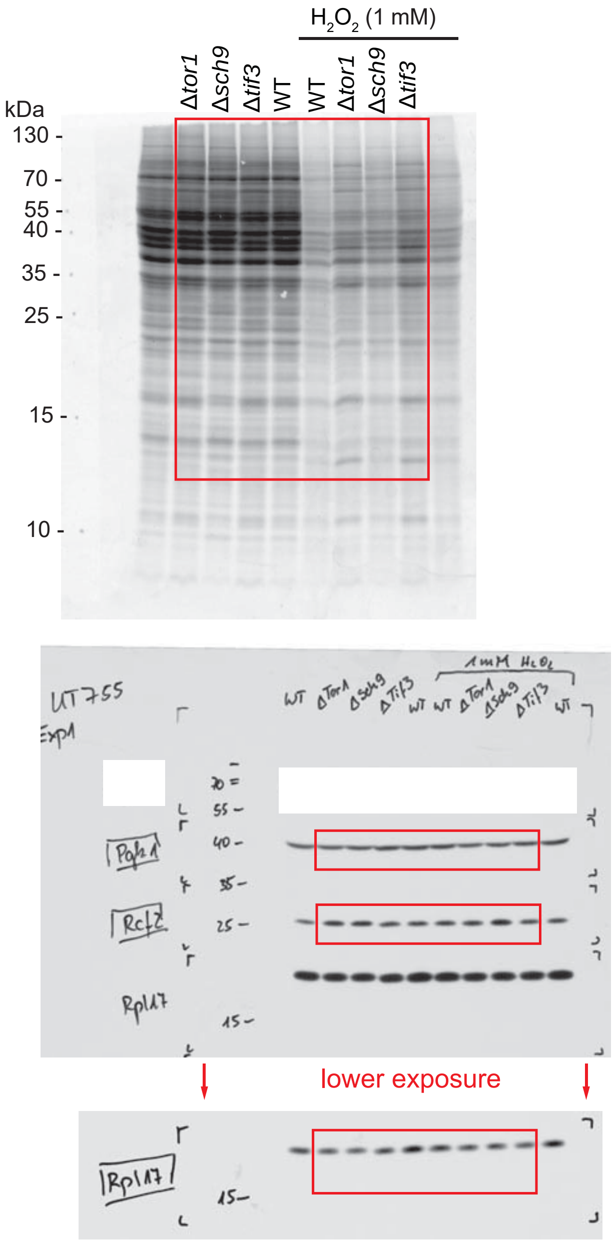

Supplementary Fig. 7e

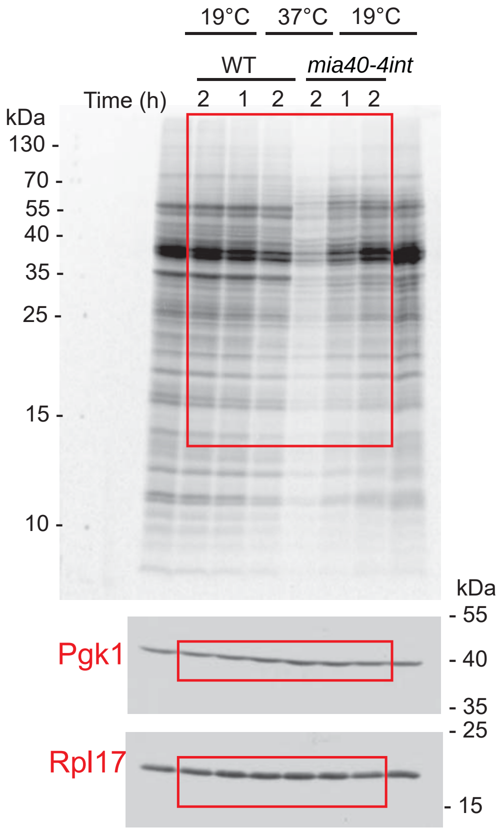

After protein transfer membranes were cut and their fragments were incubated with antibodies against indicated proteins.

SUPPLEMENTARY REFERENCES

1. Hebert, A. S. *et al.* The one hour yeast proteome. *Mol Cell Proteomics* **13**, 339-347 (2014).
2. Paulo, J. A., O'Connell, J. D., Gaun, A. & Gygi, S. P. Proteome-wide quantitative multiplexed profiling of protein expression: carbon-source dependency in Saccharomyces cerevisiae. *Mol Biol Cell* **26**, 4063-4074 (2015).
3. Kulak, N.A., Pichler, G., Paron, I., Nagaraj, N. & Mann, M. Minimal, encapsulated proteomic-sample processing applied to copy-number estimation in eukaryotic cells. *Nat Methods* **11**, 319-U300 (2014).
4. Crooks, G. E., Hon, G., Chandonia, J. M. & Brenner, S. E. WebLogo: a sequence logo generator. *Genome Res* **14**, 1188-1190 (2004).
5. Amunts, A. *et al.* Structure of the yeast mitochondrial large ribosomal subunit. *Science* **343**, 1485-1489 (2014).
6. Garreau de Loubresse, N. *et al.* Structural basis for the inhibition of the eukaryotic ribosome. *Nature* **513**, 517-522 (2014).
7. Llacer, J. L. *et al.* Conformational Differences between Open and Closed States of the Eukaryotic Translation Initiation Complex. *Mol Cell* **59**, 399-412 (2014).
8. Sievers, F. *et al.* Fast, scalable generation of high-quality protein multiple sequence alignments using Clustal Omega. *Mol Syst Biol* **7**, 539 (2011).
9. Jin, K. *et al.* Yeast mitochondrial protein-protein interactions reveal diverse complexes and disease-relevant functional relationships. *J. Proteome Res.* **14**, 1220-1237 (2015).
